# Cardiometabolic regulation by adipocyte-derived leptin and the brain melanocortin system

**DOI:** 10.1042/CS20250039

**Published:** 2026-06-24

**Authors:** John E. Hall, Ana C.M. Omoto, Jussara M. do Carmo, Alexandre A. da Silva, Alan J. Mouton, Zhen Wang, Michael E. Hall

**Affiliations:** 1 Department of Physiology and Biophysics, University of Mississippi Medical Center, Jackson, MS, U.S.A.; 2 Mississippi Center for Obesity Research, University of Mississippi Medical Center, Jackson, MS, U.S.A.; 3 Cardiorenal and Metabolic Diseases Research Center, University of Mississippi Medical Center, Jackson, MS, U.S.A.; 4 Mississippi Center for Clinical and Translational Research, University of Mississippi Medical Center, Jackson, MS, U.S.A.; 5 Department of Medicine, University of Mississippi Medical Center, Jackson, MS, U.S.A.

**Keywords:** acute kidney injury, diabetes mellitus, heart failure, hypertension, obesity, sympathetic nervous system

## Abstract

Tonic activation of central nervous system (CNS) leptin receptors (LepRs) and melanocortin 4 receptors (MC4Rs) is critical for maintaining normal cardiometabolic function. Deficiency of CNS LepR or MC4R signaling causes severe obesity and is accompanied by multiple metabolic abnormalities including insulin resistance, glucose intolerance, and hyperlipidemia that are only partially explained by obesity. Defective LepR and MC4R signaling also causes dysfunction of the sympathetic nervous system (SNS) and blood pressure (BP) regulation. Hyperleptinemia and activation of the CNS melanocortin system in obesity are important compensatory mechanisms that attenuate abnormalities of glucose and lipid metabolism but may also contribute to SNS activation and increased BP. Despite potentially adverse effects of increases in SNS activity and BP, pharmacological activation of brain LepRs and MC4Rs may provide an important therapeutic approach for protecting target organs, such as the heart, kidneys, and brain, from ischemic injury by improving mitochondrial function and ATP production. However, additional preclinical studies are needed to address mechanistic questions before clinical studies are begun to test the effectiveness of leptin and MC4R agonists for treating people with ischemic injury of target organs.

## Introduction

Adipocytes are much more than energy storage depots. They also serve important endocrine functions, secreting substances that influence blood pressure (BP) and other cardiovascular functions, as well as multiple metabolic functions. Leptin, the first adipocyte-derived hormone discovered, is generally secreted in proportion to adiposity although additional factors associated with feeding (e.g. insulin and glucose) also stimulate acute increases in plasma leptin that usually peak several hours after a meal [1]. Since the discovery of leptin, many ‘adipokines’, hormones, and other factors have been found that enable adipocytes to communicate with other tissues in the body. In this review, we focus mainly on the role of adipocyte-derived leptin in cardiometabolic regulation and one of its key central nervous system (CNS) mediators, the melanocortin system.

The importance of leptin as a feedback regulator of energy balance is clearly demonstrated by the finding that loss of function mutations of the leptin gene or leptin receptors (LepRs) cause severe, early onset obesity due to increased food intake and reduced energy expenditure in experimental animals and humans [2]. Although leptin’s powerful role in regulating energy balance was the basis for its discovery, many additional functions of leptin have been revealed over the past three decades [3]. For example, leptin has powerful antidiabetic effects, independent of changes in food intake, due to suppression of glucose production by the liver and stimulation of glucose uptake and utilization by peripheral tissues such as skeletal muscle, heart, and adipose tissue [3–5].

Because leptin binding sites were found in regions of the brain that are important for cardiovascular regulation, as well as for energy balance, we and others directed attention to its potential role in regulating sympathetic nervous system (SNS) activity and cardiovascular function. These studies revealed that leptin stimulates SNS activity and may be an important link between obesity and hypertension [6–8].

**Figure 1:**
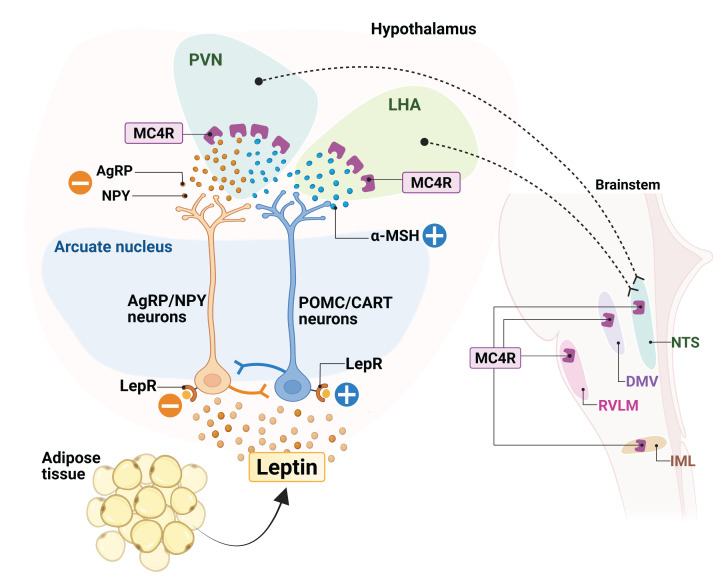
Leptin stimulates POMC/CART neurons and inhibits AGRP/NPY neurons Control of cardiometabolic function by two general types of neurons in the arcuate nucleus of the hypothalamus: (1) POMC/cocaine- and amphetamine-regulated transcript (CART) neurons that, when stimulated, decrease food intake, increase sympathetic activity, and increase energy expenditure; and (2) AgRP/neuropeptide Y (NPY) neurons that, when stimulated, increase food intake and decrease energy expenditure. POMC/CART and AgRP/NPY neurons send projections to second-order neurons in the paraventricular nucleus (PVN) and lateral hypothalamic area (LHA) where they release α-MSH and AgRP, respectively. α-MSH stimulates MC4R whereas and AgRP inhibits MC4R. Neurons in the PVN and LHA send projections to the brainstem neurons that, along with hypothalamic neurons, regulate cardiometabolic functions. LepR, leptin receptors; NTS, nucleus tractus solitarius; DMV, dorsal motor nucleus of the vagus; RVLM, rostral ventrolateral medulla; IML, intermediolateral nucleus.

The CNS melanocortin system mediates a major part of leptin’s cardiometabolic actions and has important leptin-independent actions on cardiovascular and metabolic functions [4,8,9]. Key components of this system include proopiomelanocortin (POMC) neurons that produce α-melanocyte stimulating hormone (α-MSH) that activates CNS melanocortin receptors, especially melanocortin 4 receptors (MC4Rs) and melanocortin 3 receptors (MC3Rs), to reduce food intake and increase energy expenditure (Figure 1). Neurons that release agouti-related peptide (AgRP) represent another component of this system. AgRP serves as an endogenous inverse agonist of MC4R and MC3R, inducing arrestin-mediated endocytosis of these receptors, suppressing their constitutive activity, and antagonizing activation of these receptors by α-MSH [10,11].

The importance of the CNS melanocortin system in regulating energy balance is also clearly demonstrated by the finding that loss of function mutations of POMC or MC4Rs, as well as overexpression of AgRP, lead to severe, early onset obesity associated with increased food intake and decreased energy expenditure, similar to the effects of deficient leptin signaling [12–14]. Conversely, activation of POMC neurons and MC4Rs induces satiety and increased energy expenditure.

Stimulation of the CNS melanocortin pathway also increases SNS activity and BP [15–17]. Moreover, activation of POMC neurons and MC4R signaling are required for many of the cardiovascular and antidiabetic effects of leptin [18–20]. This review of leptin and the CNS melanocortin system is organized around three short stories: (1) the interaction of leptin and the CNS melanocortin system in stimulating sympathetic activity and elevating BP in obesity, (2) the role of the leptin–melanocortin system in regulating glucose metabolism, and (3) the potential role of activating brain LepRs and MC4Rs as a therapeutic target to facilitate recovery from ischemic injury of tissues such as the heart and kidneys.

## Role of leptin and CNS melanocortins in blood pressure regulation and obesity-induced hypertension

### Leptin activates the sympathetic nervous system

Multiple studies in rodents, rabbits, and humans indicate that leptin administration increases sympathetic nerve activity (SNA). For example, *acute* infusion of leptin in rodents increases SNA in brown adipose tissue (BAT), skeletal muscle, adrenal glands, and kidneys [21]. In non-obese healthy men, acute hyperleptinemia increases muscle SNA [22]. Although the effect of *chronic* hyperleptinemia on SNA has not, to our knowledge, been reported, there is a positive association between plasma leptin concentration and muscle SNA activity in humans [23]. Moreover, leptin deficiency in adult men and women is associated with sympathetic dysfunction, postural hypotension, and normal or reduced BP despite severe obesity that usually increases SNA and BP [24]. Similar results have been reported in male leptin-deficient ob/ob mice that have lower BP than their lean controls despite severe obesity [25].

#### Potential sex differences in the effect of leptin on sympathetic activity

There is evidence for sex differences in the acute effects of leptin on sympathetic activity. For example, Shi and Brooks [26] reported that intracerebroventricular (ICV) leptin administration for 2 hours increased lumbar, splanchnic, and renal SNA similarly in anesthetized male and proestrus female rats. Leptin administration also increased splanchnic SNA and heart rate (HR) in males and in females throughout the estrus cycle. However, in female rats, the stimulatory effects of leptin on lumbar and renal SNA were observed only during proestrus and in estrogen-treated ovariectomized rats, but not in untreated ovariectomized or diestrus rats [26]. Thus, in female rats, leptin may require proestrus-levels of estrogen for some, but not all, of its sympathoexcitatory effects. Whether this is true for pre-menopausal or post-menopausal women has not, to our knowledge, been tested. It is also unclear whether these sex differences in the acute effects of leptin on SNA are sustained chronically and whether anesthesia may differentially influence the effect of leptin on SNA in males and females. We previously reported that although unanesthetized female rats had lower BP at baseline compared to males, ICV infusion of leptin for 7 days caused similar increases in BP and HR as well as similar metabolic effects in male and female rats [27]. Although these findings provide no evidence for major sex differences in leptin’s chronic brain-mediated cardiovascular or metabolic actions in conscious rats, additional studies are clearly needed, especially in humans, to better understand the potential importance of sex differences in leptin’s cardiometabolic actions.

#### Acute and chronic effects of leptin on blood pressure

Although leptin increases SNA in males (humans and rodents) as well as in female rodents with proestrus estrogen levels, acute injections of leptin often have little effect on BP in humans or in rodents, perhaps due partly to counterbalancing vasodilator effects of leptin-mediated nitric oxide (NO) released from endothelial cells [28]. However, chronic leptin infusions to produce hyperleptinemia comparable to that found in obesity causes slowly developing, sustained elevations of BP and HR in male and female rodents despite weight loss, which usually decreases BP and HR [6,27]. Also, transgenic overexpression of the leptin gene in mice increases BP despite reducing food intake and body weight [29].

Increases in BP during chronic hyperleptinemia are mild and require several days to occur, consistent with mild increases in SNA sufficient to increase renal sodium reabsorption but not to directly cause vasoconstriction. Combined α- and β-adrenergic receptor blockade completely abolished leptin-mediated chronic increases in BP in male rats, indicating that they were due to SNS activation; in fact, after adrenergic blockade, leptin infusion lowered BP and HR, perhaps due to weight loss and stimulation of NO [30]. When NO synthesis was inhibited, the chronic hypertensive and tachycardic effects of leptin were greatly amplified despite reductions of food intake and weight loss [31]. Since obesity is often associated with endothelial cell dysfunction, reduced NO bioavailability, and resistance to leptin’s anorexic effects, the effect of leptin to increase BP may be enhanced in obesity if stimulation of SNA is preserved, as previously reported in rodents [32].

In humans, the limited studies previously conducted have generally not shown significant increases in BP during leptin administration at therapeutic doses. For example, administration of recombinant leptin for 12 weeks did not raise BP in obese men and women, although BP was not a primary outcome in these studies and the doses of leptin used also failed to alter body weight [33]. In people with lipodystrophy, many of whom already have elevated BP, leptin administration does not further increase BP [34]. However, leptin therapy for people with lipodystrophy also causes important metabolic benefits including reductions in ectopic fat in organs such as the kidneys and liver and improved insulin sensitivity that could offset potential hypertensive effects of leptin-mediated increases in SNA. Thus, while leptin administration increases SNA and BP in rodents, the available evidence in humans suggests that therapeutic doses of leptin may increase SNA, but not enough to significantly raise BP. In fact, the beneficial metabolic effects of chronic leptin administration may lower BP in some individuals, such as those with lipodystrophy [34].

### Potential role of leptin in obesity hypertension

Although therapeutic doses of leptin do not always elevate BP, there is evidence that leptin may contribute to SNS activation and increased BP in obesity. For example, administration of a LepR antagonist into the lateral ventricles of mice with dietary-induced obesity reduced BP and HR [35]. Antagonism of LepRs in obese rabbits fed a high-fat diet also reduced BP and renal SNA [36]. Leptin-deficient ob/ob mice, despite having severe obesity, insulin resistance, hyperinsulinemia, and dyslipidemia, had lower BP than lean control mice [25]. Moreover, leptin replacement in ob/ob mice increased BP despite weight loss [29]. In mice lacking LepRs, re-expression of LepRs only in the dorsomedial hypothalamus (DMH) caused marked increases in BP and HR [35]. Taken together, these observations suggest that hyperleptinemia may be an important link between obesity, increased SNA, and hypertension in obese rodents and rabbits.

In humans, however, the role of leptin in mediating obesity-induced hypertension is uncertain. Children and adult men and women with leptin deficiency due to gene mutations exhibit normal or reduced BP compared to weight-matched controls [24,35]. This finding is similar to the effects of leptin deficiency on BP in rodents. Also, men and women with leptin gene mutations exhibit postural hypotension, decreased renin–angiotensin–aldosterone system (RAAS) responses to upright posture, and decreased BP responses to cold pressor stimuli, indicating autonomic insufficiency [24]. Studies that have included a small number of adults with leptin gene mutations and long-standing obesity have reported that these people may have hypertension that is exacerbated by short-term (1 week) leptin treatment [37]. However, with long-term (7–14 months) leptin treatment and weight loss, BP decreased in some people [37], illustrating that the complex metabolic and SNS effects of leptin may have opposing effects on BP and offset each other in some cases.

Thus, current data, although sparse, suggest that leptin increases SNA in humans and that children as well as adult men and women with leptin gene mutations and leptin deficiency may not be hypertensive or have increased sympathetic activity despite severe obesity and metabolic abnormalities that usually increase BP. These observations, together with similar findings in rodents, are consistent with the concept that leptin may contribute to obesity-induced SNS activation and hypertension.

As discussed previously, acute studies in rodents suggest that there may be sex differences in the role of leptin in sympathetic activation and potentially in obesity-induced hypertension. Plasma leptin concentration is generally higher in women than in men when equated for body weight [38]. However, despite higher plasma leptin levels, SNA and BP in pre-menopausal women is often lower than in age-matched men, although these differences disappear after age 40 to 49 years [39]. Whether these differences in BP and SNA are related to differences in leptin sensitivity or to other factors, such as leptin-independent effects of excess visceral fat is unclear. After menopause, women often experience increases in BP and a higher prevalence of hypertension compared to men of the same age [40]. Although postmenopausal hypertension is likely multifactorial, increased visceral adiposity appears to play an important role [41].

**Figure 2:**
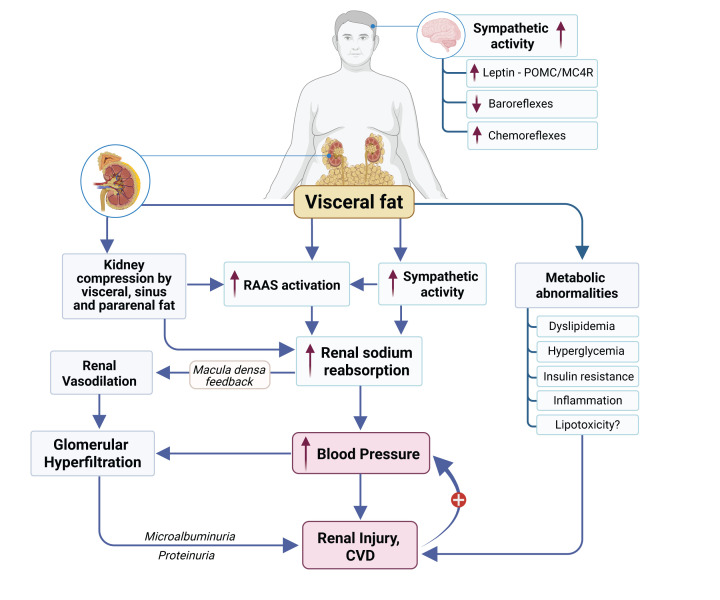
Summary of mechanisms that contribute to obesity-induced hypertension, renal injury, and cardiovascular disease (CVD) Summary of mechanisms that contribute to obesity-induced hypertension, renal injury, and cardiovascular disease. POMC, pro-opiomelanocortin; MC4R, melanocortin receptor; RAAS, renin–angiotensin–aldosterone system.

Chronic increases in visceral adiposity increase BP through additional factors besides leptin and SNS activation, including activation of the RAAS and compression of the kidneys by increased visceral, pararenal, perirenal, and renal sinus fat [18,42] (Figure 2). Obesity, via increases in BP, glomerular hyperfiltration, and metabolic abnormalities, is also a major driver of chronic kidney disease that, over time, may contribute to gradual increases in BP and salt sensitivity of BP [18,42–44]. The non-leptin mediated effects of obesity may eventually cause increased BP, even in adults with defective LepR signaling and autonomic dysfunction.

### CNS neurons and molecular pathways responsible for cardiometabolic actions of leptin

LepRs are expressed in many areas of the brain, including the ventromedial hypothalamus, arcuate nucleus (ARC), and DMH, as well as in vasomotor centers of the brainstem and spinal cord intermediolateral nucleus (IML). Although the CNS centers that mediate leptin’s action on SNA and cardiometabolic functions have not been precisely mapped, hypothalamic centers and extra-hypothalamic regions such as the brainstem and subfornical organ have been implicated and discussed in previous reviews [45,46]. In this review we focus mainly on POMC neurons that are especially important for mediating leptin’s actions on SNA and cardiovascular regulation.

**Figure 3:**
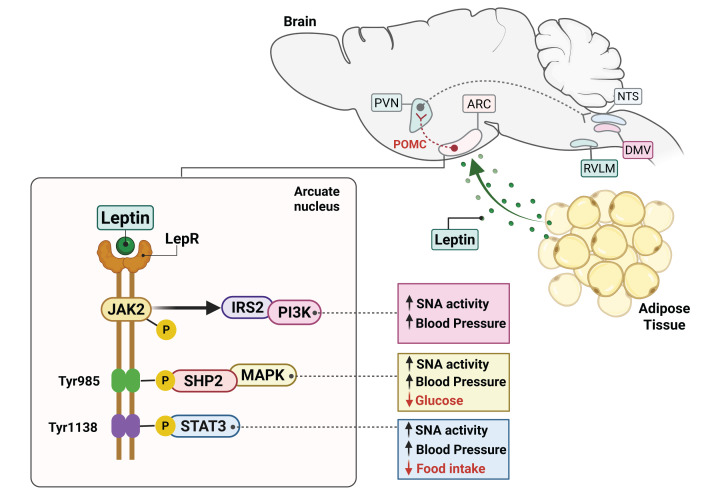
Leptin signaling pathways in neurons Leptin signaling pathways in neurons and their effects on SNA, BP, blood glucose levels, and food intake. Stimulation of LepR activates JAK2 tyrosine kinase, causing autophosphorylation of tyrosine residues on JAK2 and phosphorylation of Tyr985 and Tyr1138. Phosphorylation of Tyr985 activates SHP2/MAPK and phosphorylation of Tyr1183 activates STAT3. All three signaling pathways appear to contribute leptin-mediated increases in SNA and BP while the SHP2/MAPK pathway contributes to reductions in blood glucose levels and the STAT3 pathway mediates most of leptin’s anorexic effects.

In POMC neurons and other neurons, LepR activation stimulates janus tyrosine kinase 2 (JAK2) activity and three intracellular signaling pathways (Figure 3) [47]. Signal transducers and activators of transcription 3 (STAT3) mediate a major part of the anorexic and energy expenditure effects of leptin [18]. Insulin receptor substrate 2 (IRS2)–phosphatidylinositol 3-kinase signaling mediates at least part of leptin’s effects on SNA and BP but is less important in regulating energy balance [48]. Src homology-2 tyrosine phosphatase–mitogen-activated protein kinase (SHP2–MAPK) signaling in the forebrain appears to be important in controlling food intake, energy balance, blood glucose, and BP [49]. Moreover, the effects of leptin on BP and glucose due to forebrain SHP2–MAPK signaling are mediated mainly by POMC neurons, whereas effects on food intake are also mediated to a great extent by other forebrain neurons [49,50].

It is possible that obesity differentially affects these three pathways and their contributions to cardiometabolic regulation, although this hypothesis has not been adequately tested. However, several studies have provided evidence that POMC neuron activation is essential for leptin’s chronic effects on regulation of glucose, renal SNA, and BP, whereas other neurons appear to be more critical for leptin’s actions on energy balance.

### Role of CNS melanocortin system in sns activation and obesity hypertension

#### Activation of POMC neurons and MC4Rs contribute to leptin’s cardiometabolic effects

The CNS melanocortin system is a key pathway for linking leptin with cardiometabolic regulation [8,9,18]. Leptin activates POMC neurons in the ARC of the hypothalamus. These neurons project to second order neurons involved in cardiometabolic regulation, such as the PVN, lateral hypothalamus, and DMH, where their axons release α-MSH, the endogenous ligand for MC4Rs. Brainstem centers involved in cardiovascular regulation, including the nucleus tractus solitarius (NTS) and dorsal motor nucleus of the vagus (DMV), also express POMC and MC4Rs [51,52]. Preganglionic sympathetic neurons in the IML of the spinal cord also contain MC4Rs. Although there is evidence that each of these neuronal centers plays a role in cardiometabolic regulation, their relative importance in mediating the actions of leptin has not been fully elucidated.

The overall importance of the CNS melanocortin pathway in regulating energy balance, however, is evident from the finding that loss of function mutations of POMC or MC4Rs cause severe hyperphagia, reduced energy expenditure, early-onset obesity, and multiple metabolic abnormalities including insulin resistance and increased risk for diabetes [53,54]. In fact, genetic variants of the CNS melanocortin pathway represent the most common cause of monogenic obesity, accounting for 3%–7% of early onset, morbid obesity in children [55–57].

Although leptin stimulates POMC neurons, deletion of LepRs specifically in POMC neurons causes only mild obesity whereas total neuronal LepR deletion induces hyperphagia and severe obesity, as does genetic deletion of MC4Rs [58–60]. These observations suggest two important points: (1) a major part of leptin’s effects on satiety and energy balance is mediated by non-POMC neurons, and (2) additional factors besides leptin play an important role in activating the POMC–MC4R pathway.

While only partially responsible for leptin’s effects on energy balance, the POMC–MC4R pathway plays a major role in mediating the actions of leptin on SNA, BP, and glucose regulation. Genetic deletion of LepRs specifically on POMC neurons completed abolished the chronic BP and antidiabetic effects of leptin in male mice but had only a modest effect on leptin’s anorexic actions [59]. Pharmacological blockade or genetic deletion of MC4Rs also eliminated the BP and antidiabetic effects of leptin in male rats [8,61].

#### Activation of the CNS melanocortin system increases SNS activity and BP

Acute ICV administration of MC4R agonists stimulates SNA in multiple tissues, including BAT, muscle, and kidneys, although the level of sympathetic activation is not sufficient to raise BP over several hours [8,62]. Similar to the effects of leptin, however, chronic ICV infusion of MC4R agonists in rodents causes a gradual, mild increase in BP despite reducing food intake and body weight [15]. The pressor response to chronic MC4R activation in male rats is abolished after combined α- and β-adrenergic blockade indicating that it is due to increased SNS activity [63]. In humans with obesity, treatment with synthetic MC4R agonists has been reported to increase BP and HR despite causing weight loss [64]. Although the sympathomimetic effects of MC4R agonists have limited their use in treating obesity, some MC4R agonists (e.g. setmelanotide) appear to have minimal pressor effects [65].

Conversely, chronic ICV administration of MC4R antagonists in male and female rodents reduces BP despite causing hyperphagia and rapid weight gain [19,66,67]. The BP lowering effects of MC4R antagonism are especially pronounced when sympathetic activity is increased such as occurs with high-fat diet-induced obesity and in spontaneously hypertensive rats (SHR) [16,66,68]. Likewise, chronic ICV infusion of AgRP, which suppresses constitutive MC4R signaling and antagonizes α-MSH activation of the receptor, reduces BP despite causing hyperphagia and obesity in rats [69].

#### Loss-of-function mutations of the POMC–MC4R system reduce SNS activity and BP despite causing severe obesity

Although mice with genetic deficiency of MC4Rs exhibit hyperphagia, severe obesity, insulin resistance and hyperinsulinemia, they are not hypertensive on normal or high salt diets [70]. In fact, MC4R deficient mice tend to have lower BP and HR compared to lean control mice despite severe obesity and hyperleptinemia [60,70]. MC4R deficient male mice also have attenuated BP responses to acute stress and their BP responses to chronic leptin infusion are completely abolished [60,71].

MC4R deficiency in male rats also abolishes the BP responses to chronic leptin infusion [72]. Some studies have reported that male and female MC4R deficient rats have slightly higher baseline BP compared to lean control rats [73,74]. However, in male and female rats and mice pharmacological blockade of MC4R reduces BP despite causing severe obesity and hyperleptinemia [66,68,73,75].

Humans (male and female) with loss of function MC4R mutations also have lower BP and HR, reduced prevalence of hypertension, and decreased 24-hour norepinephrine excretion compared to control subjects, despite severe obesity and associated metabolic disorders [64,76]. In obese people with MC4R mutations there is also an inverse relationship between obesity and muscle SNA whereas people with functional MC4Rs exhibit a positive relationship between obesity and muscle SNA [77]. Reductions in SNA and BP were observed in severely obese people with MC4R mutations, compared to control subjects, despite large increases in plasma leptin levels and multiple metabolic abnormalities (e.g. insulin resistance and hyperinsulinemia) that would usually tend to increase BP. These findings suggest that normal sympathetic tone depends on active MC4R signaling and that a functional POMC–MC4R system may be necessary for obesity and hyperleptinemia to increase SNS activity and BP, at least prior to development of obesity-induced injury of target organs such as the kidneys.

#### The CNS melanocortin system is activated by factors other than hyperleptinemia and obesity

Although many of the factors that stimulate POMC neurons, such as insulin, amylin, glucose, and amino acids, are associated with the fed state, additional factors such as nicotine and increased body temperature may also activate POMC neurons [52]. The CNS melanocortin system also regulates BP independent of hyperleptinemia and obesity. For example, MC4R blockade markedly reduced BP in SHR, a non-obese model of hypertension with normal plasma leptin levels and increased SNS activity [68]. MC4R antagonism also reduced BP in obese Zucker rats with disrupted LepR signaling [78] and attenuated the BP effects of nesfatin-1, neuronostatin, and amphetamine [79]. MC4R blockade also attenuated hypertension caused by NO synthesis inhibition but did not lower BP in angiotensin II hypertension that is associated with decreased renal SNA [80]. Thus, MC4R blockade appears to have a tonic influence on SNA and BP in normal animals and reduces BP to an even greater extent when SNA is increased. Whether MC4R activation is driving increased SNA in these conditions, or playing a permissive role via interactions with other factors, is uncertain.

In humans, MC4R may also contribute to increased SNA in stress conditions, independent on changes in plasma leptin levels. For example, the effect of hypoxic stress to increase muscle SNA is attenuated in people with MC4R deficiency [77]. Thus, the POMC–MC4R pathway may contribute to SNS activation during acute stress as well as during chronic increases in SNS activity caused by obesity or other stresses. Taken together, these observations suggest that this system may play a more fundamental role controlling SNS activity and BP than previously appreciated.

#### The CNS melanocortin system plays a role in controlling respiratory function

LepRs and MC4Rs are located in brainstem neurons involved in controlling respiration. Genetic deficiency of leptin or MC4Rs, or pharmacological blockade of MC4Rs, attenuated the ventilatory response to hypercapnia in rodents [81,82]. The impairment of ventilatory responses in leptin deficient mice cannot be explained by obesity since lean ob/ob mice that were weight matched with lean control mice by pair feeding had similar impairment of the ventilatory responses to hypercapnia as observed in obese ob/ob mice [83]. Mice that overexpress the agouti protein, which inhibits MC4Rs, also have impaired ventilatory responses to CO_2_ compared to weight matched controls [84]. Activation of POMC neurons and MC4Rs is also essential for the effects of leptin to stimulate the ventilatory response to hypercapnia [81]. MC4Rs in the carotid body may also enhance chemosensory afferent drive and chemoreflex-evoked sympathetic and ventilatory drive [85]. Thus, the CNS POMC–MC4R pathway appears to be essential for normal respiratory responses to hypercapnia as well for leptin’s stimulatory effects on ventilation and SNA.

#### CNS centers and signaling pathways that mediate autonomic and BP effects of MC4R activation

MC4Rs are expressed in multiple CNS centers that have been implicated in autonomic regulation, including the PVN, lateral hypothalamus, amygdala, NTS, DMV, and preganglionic sympathetic neurons of the IML. The PVN has the highest density of MC4Rs and acute studies indicate that microinjection of an MC4R agonist into the PVN increases renal SNA and BP [86]. Also, blockade of MC4Rs in the PVN abolished the acute effect of hyperinsulinemia to increase lumbar SNS activity [87]. MC4Rs on POMC neurons may also modulate POMC activity and autonomic function. Rescue of MC4R function specifically in POMC neurons of male mice with whole-body MC4R deficiency partially restored the BP response to acute stress, suggesting that MC4R may serve to autopotentiate POMC neuronal activity [60]. Although MC4Rs on cholinergic preganglionic parasympathetic and sympathetic neurons have been suggested to contribute to obesity hypertension [88], the specific neurons that mediate the effects of MC4R on SNA and BP in obesity and other chronic conditions are still largely unknown.

Additional downstream signaling pathways for MC4Rs have been proposed, although their importance in cardiometabolic regulation is still uncertain. For example, MC4R activation increased brain-derived neurotrophic factor (BDNF) protein in the DMV of rats, and the acute orexigenic effect of an MC4R antagonist injected in the 4^th^ ventricle was blocked by co-administration of BDNF [89]. Reductions in food intake and increases in BP after acute injections of an MC4R agonist were attenuated by prior CNS administration of an anti-BDNF antibody [90]. Other candidates, such as corticotrophin-releasing hormone, melanin-concentrating hormone, and oxytocin have also been suggested to contribute to MC4R’s actions on appetite [91]. However, it is still unclear whether any of these potential mediators contribute to the chronic effects of MC4R activation on SNA and BP.

MC4Rs are G protein-coupled receptors that increase cAMP phosphorylation and protein kinase A [91]. One factor that amplifies MC4R signaling is the melanocortin receptor accessory protein 2 (MRAP2), which is co-expressed with MC4Rs [92]. MRAP2 increases MC4R signaling by enhancing of G-protein coupling and reducing internalization of MC4Rs. Although genetic deletion of MRAP2 in MC4R neurons did not affect baseline BP, it decreased HR, baroreflex sensitivity, and tonic renal SNA. Loss of MRAP2 also blunted the acute effects of MC4R activation on food intake, SNA, HR, and BP as well as the development of obesity-induced hypertension in mice [93]. Thus, MRAP2 appears to play a key role in regulating MC4R signaling and cardiometabolic function.

Although MC4R is known to activate the Gs protein that increases intracellular cAMP signaling, more recent evidence indicates that MC4R/MRAP2 also signals via Gq [94]. Mutations of MC4R that selectively interfere with Gq (but not Gs) coupling are associated with hyperphagia and obesity [95]. These findings suggest that targeting MC4R/MRAP2 signaling to activate satiety pathways (Gq signaling) while avoiding activation of the Gs–cAMP pathway responsible for sympathetic stimulation may represent a therapeutic strategy for treating obesity without the detrimental effect of increased BP. However, since the effects of MC4R on energy expenditure and glucose metabolism appear to be primarily mediated by activation of Gs [96], a biased MC4R ligand that selectively activates Gq might also be expected to lack these beneficial effects.

#### Role of the melanocortin 3 receptor in regulation of BP and energy balance

Although we have focused mainly on the MC4R as a key component of the CNS melanocortin system, the MC3R also plays a role in energy homeostasis and many of the agonists and antagonist of MC4R that we discussed also activate or inhibit the MC3R. The MC3R is also a G protein-coupled receptor that is stimulated by α-MSH and localized in the same regions of the CNS as MC4R [97]. However, the MC3R is also expressed in the kidneys where it is activated by γ-MSH and enhances natriuresis [98]. In contrast to MC4R activation that tends to increase BP, MC3R activation is associated with natriuresis and protection against salt-sensitive hypertension; although mice with MC3R deficiency have normal BP when salt intake is normal, they develop hypertension when ingesting a high salt diet, partly due to defective γ-MSH signaling in the kidneys [99].

The effects of MC3R activation on energy balance also differ from those of MC4R. Whereas MC4R activation reduces food intake and increases sympathetic activity and energy expenditure, MC3R activation has a more subtle role in energy homeostasis. Genetic deficiency of MC3R on rodents also leads to increased adiposity, although the obesity is less severe than observed with MC4R deficiency and occurs later in life [100]. MC3R activation alters nutrient partitioning to favor reduction of fat mass, increasing energy efficiency and influencing feeding rhythms rather than having a major effect on total food intake [100]. Recent preliminary studies indicate that dual MC3R/MC4R activation may cause substantial weight loss in non-human primates without increasing BP, as often occurs with chronic administration of MC4R agonists [101].

### Does the leptin–melanocortin system activate the renin–angiotensin–aldosterone system?

Some studies suggest that obesity may be an important driver of inappropriately elevated plasma aldosterone concentration (e.g. idiopathic hyperaldosteronism), especially when considering that obesity is generally associated with volume expansion and elevated BP that would normally suppress renin and aldosterone secretion [102]. Although the mechanisms responsible for inappropriate aldosterone section in obesity have not been fully elucidated, adipocyte-derived factors, including leptin, have been suggested to be involved [103]. However, chronic leptin infusion in male rodents did not significantly increase PRA or plasma aldosterone concentration [6]. In contrast, studies in female rodents suggest that leptin, circulating in the blood or released from pararenal fat or from adipose tissue near the adrenal glands, may directly stimulate adrenal CYP11B2 (aldosterone synthase) expression and aldosterone secretion [104]. The stimulatory effect of leptin on aldosterone was observed only in female rodents and there have been no studies, to our knowledge, indicating a direct stimulatory effect of hyperleptinemia on aldosterone secretion in humans, male or female.

Although plasma renin and aldosterone levels may not be substantially elevated in many obese people (male or female), blockade of mineralocorticoid receptors (MR) is widely recognized to be effective in lowering BP in people with obesity and treatment resistant hypertension [18,105]. The antihypertensive benefit of MR blockade in men and women with obesity occurs over a wide range of plasma renin and aldosterone concentrations and appears to be related, at least in part, to aldosterone-independent MR activation [42,105].

## Antidiabetic actions of leptin and activation of the CNS melanocortin system

Although hyperleptinemia and MC4R activation may increase SNA and BP in obesity, normal activity of the leptin–CNS melanocortin system has major beneficial effects on glucose homeostasis and energy balance. Deficiencies of leptin or POMC neuronal activation are associated with insulin resistance, glucose intolerance and diabetes mellitus, effects that are ameliorated or reversed with replacement therapy [4,106]. For example, in humans with lipodystrophy and leptin deficiency, treatment with recombinant leptin reverses insulin resistance and diabetes [107]. Rodents with loss-of-function mutations of the leptin gene or LepR also have insulin resistance, hyperinsulinemia, and glucose intolerance that are not fully normalized by pair feeding to prevent the hyperphagia and obesity associated with defective leptin signaling [4,108]. Likewise, loss of function mutations of POMC or MC4R in humans and rodents are associated with insulin resistance, glucose intolerance and a propensity to develop diabetes [4,106].

Administration of exogenous leptin exerts powerful actions on glucose regulation, markedly increasing glucose uptake and metabolism in multiple tissues, while reducing hepatic glucose production and enhancing insulin sensitivity [109]. Pair feeding to produce similar reductions in food intake caused by leptin administration does not recapitulate these effects on glucose regulation. Moreover, leptin administration completely normalizes plasma glucose in rodents treated with streptozotocin (STZ) to induce type 1 diabetes (T1D) despite plasma insulin levels that are barely detectable [110]. Thus, the antidiabetic effects of leptin are at least partly independent of weight loss, plasma insulin levels, or increased insulin sensitivity. As discussed later, the antidiabetic actions of leptin depend, to a great extent, on a normally functioning CNS melanocortin system, although POMC–MC4R activation cannot fully recapitulate the glucose lowering effects of hyperleptinemia.

### Partial resistance to the antidiabetic effects of leptin in obesity

Although leptin administration induces powerful antidiabetic effects in conditions of leptin deficiency (e.g. leptin gene mutations, congenital lipodystrophy, or T1D), the antidiabetic effects of administering additional leptin are diminished when leptin levels are already elevated, as occurs in common obesity [111,112]. This ‘resistance’ to the metabolic effects of leptin in common obesity may be partly due to diminished leptin transport across the blood brain barrier (BBB) [113]. Leptin crosses the BBB via a saturable transporter that involves the short form of the LepR [113]. Common obesity and high leptin levels are also associated with increased levels of suppressor of cytokine signaling 3, protein tyrosine phosphatase 1B, and mechanistic Target of Rapamycin in the hypothalamus that attenuate LepR signaling [47,114]. Despite partial leptin resistance in common obesity, genetic or pharmacological blockade of the leptin–melanocortin system worsens obesity and metabolic dysregulation indicating that this system retains, albeit diminished, endogenous antidiabetic and anti-obesity actions [115,116]. Although leptin’s metabolic effects are blunted in obesity, its stimulatory actions on SNA and BP appear to be preserved, suggesting ‘selective’ leptin resistance [117,118].

### Activation of CNS leptin receptors has powerful antidiabetic effects

**Figure 4:**
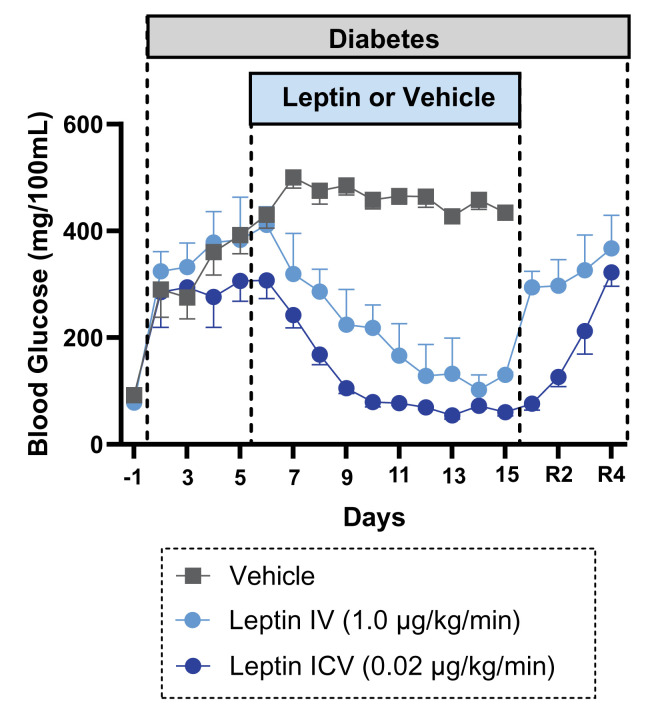
Antidiabetic effects of chronic IV or ICV leptin infusion Effect of intravenous (IV, 1.0 μg/kg/min) or intracerebroventricular (ICV, 0.02 μg/kg/min) leptin infusion, or saline vehicle infusion for 10 days on blood glucose concentration in rats treated with STZ to induce T1D mellitus. (Redrawn from data in [110])

Although leptin has direct effects on the liver and kidneys to reduce gluconeogenesis and on other peripheral tissues (e.g. skeletal muscle, heart, adipose) to increase glucose uptake and utilization, its chronic antidiabetic effects are mediated to a great extent by activation of LepRs in the CNS [4,109]. In insulin-deficient T1D rats, chronic ICV leptin administration, at a rate that did not increase plasma leptin levels, produced greater and more rapid reductions of blood glucose concentration when compared with intravenous leptin infusion at a 50-fold greater dose (Figure 4) [110]. Pair feeding to produce similar decreases in body weight as observed during leptin infusion produced only small decreases in plasma glucose levels compared with those observed during ICV leptin infusion, indicating that the antidiabetic actions of leptin were not due primarily to weight loss [110].

Further support for a key role of CNS LepRs in mediating most of the long-term effects of leptin on glucose homeostasis comes from the finding that selective deletion of LepRs in neuronal cells impairs glucose regulation, leading to insulin resistance and hyperinsulinemia despite large increases in plasma leptin concentration and intact peripheral LepR signaling [58]. Also, selective restoration of LepRs in the ARC of the hypothalamus, or specifically in POMC neurons, can normalize blood glucose in LepR-deficient (db/db) mice [119,120]. Thus, leptin has powerful CNS-mediated antidiabetic effects that are independent of insulin or reductions in food intake and body weight.

### Autonomic nervous system activation is not required for the chronic antidiabetic actions of leptin

**Figure 5:**
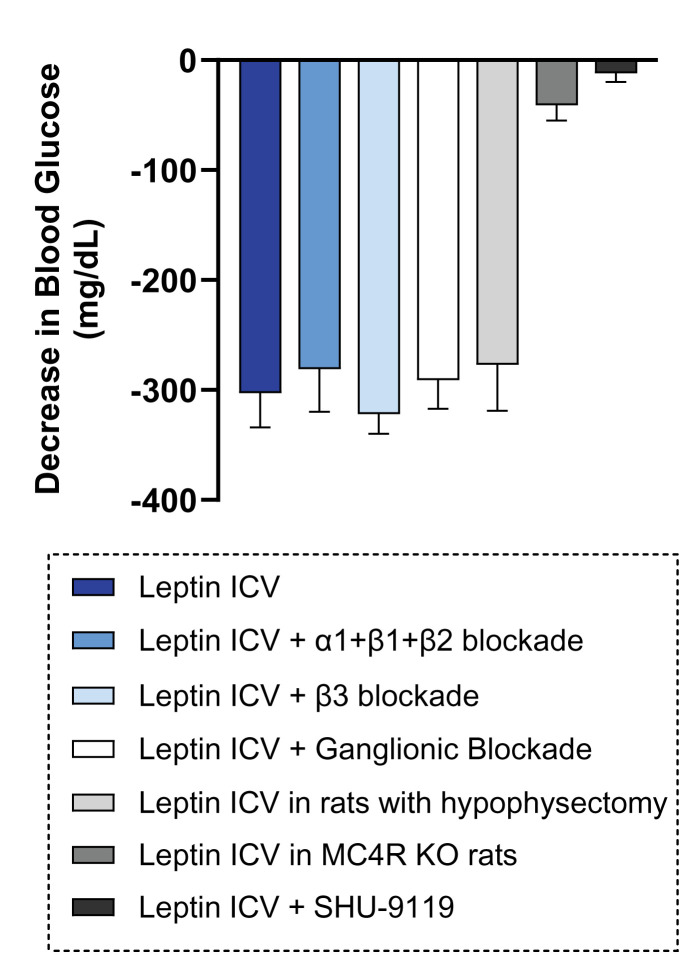
CNS-mediated chronic antidiabetic effects of leptin Decreases in blood glucose concentration after chronic intracerebroventricular (ICV, 0.02 μg/kg/min) leptin infusion. Adrenergic blockade (α1, β1, β2, and β3), ganglionic blockade, and hypophysectomy did not significantly attenuate the reductions in blood glucose concentration during ICV leptin infusion in rats treated with STZ to induce T1D mellitus. However, the antidiabetic effect of ICV leptin infusion was almost completely abolished in MC4R knock out rats and in rats treated with the MC4R antagonist SHU-9119. (Redrawn from data in [19,61,110,125,128])

A key mechanism by that the CNS communicates with peripheral tissues is the autonomic nervous system, which contributes importantly to leptin’s cardiovascular actions [42,121,122]. Support for a role of the autonomic nervous system in mediating the antidiabetic actions of leptin is the finding that adrenergic receptor antagonism or skeletal muscle denervation attenuated increased glucose uptake in skeletal muscle after *acute* leptin injections or ICV infusion of leptin [123,124]. However, combined α- and β-adrenergic blockade did not significantly attenuate the *chronic* antidiabetic effects of chronic ICV leptin infusion, which completely normalized blood glucose in T1D rats (Figure 5) [110]. Ganglionic blockade or hepatic vagotomy also did not attenuate the chronic blood glucose-lowering effects of ICV leptin infusion in insulin-deficient diabetic rats [125]. These observations indicate that changes in autonomic activity, including sympathetic nervous stimulation of tissue glucose uptake and vagal/parasympathetic inhibition of glucose output by the liver, cannot explain the powerful CNS-mediated chronic antidiabetic effects of leptin.

### Activation of the pituitary–adrenal axis is not required for the chronic antidiabetic actions of leptin

Leptin has important effects on the hypothalamic–pituitary–adrenal (HPA) axis. Some studies suggest that decreased leptin levels in T1D activate the HPA axis and that restoration of plasma leptin may restore normoglycemia mainly by suppression of the HPA axis, leading to reductions in pituitary secretion of adrenocorticotrophic hormone and adrenal secretion of glucocorticoids. In support of this concept, infusions of glucocorticoids have been reported to block the antidiabetic effects of leptin [126,127]. However, other studies indicate that HPA suppression may not be required for the chronic CNS-mediated antidiabetic actions of leptin. For example, hypophysectomy did not attenuate the chronic effects of ICV leptin infusion on glucose metabolism in diabetic or non-diabetic rats (Figure 5) [128]. Morton et al. [129] also demonstrated that adrenalectomy-induced glucocorticoid deficiency or pharmacological glucocorticoid receptor blockade did not reduce blood glucose levels in T1D rats; they also found that the glucose-lowering effect of ICV leptin infusion was not attenuated by systemic administration of corticosterone at a dose that maintained elevated plasma levels characteristic of insulin-deficient diabetes. Thus, the CNS-mediated antidiabetic effects of leptin in T1D may not require changes in pituitary hormone secretion or reductions in adrenal secretion of glucocorticoids.

### Role of the brain melanocortin system in mediating leptin’s effects on glucose homeostasis

The brain melanocortin system contributes importantly to normal glucose regulation and plays a key role in the chronic antidiabetic effects of leptin. Pharmacological antagonism or genetic deficiency of MC4R or POMC causes insulin resistance, hyperinsulinemia and glucose intolerance despite large increases in secretion of leptin [4,106]. Although hyperphagia and obesity contribute to glucose dysregulation associated with MC4R or POMC deficiency, restoring MC4R expression specifically in the lateral hypothalamus attenuates glucose intolerance in obese MC4R-deficient mice without affecting body weight or plasma insulin levels [130]. Also, pharmacological activation of brain MC4R increases insulin sensitivity and reduces plasma insulin and glucose concentrations even in the absence of weight loss [4,131]. These and many other studies highlight an important role of the CNS melanocortin system for normal glucose homeostasis via mechanisms that are at least partly independent of changes in food intake and body weight.

#### Activation of POMC neurons and stimulation of MC4R are required for leptin’s antidiabetic actions

Deletion of LepRs specifically in POMC neurons markedly attenuated leptin’s effect to reduce plasma insulin and glucose levels as well as BP in non-diabetic mice [59]. Moreover, functional MC4Rs are required for leptin’s antidiabetic effects in non-diabetic rodents as well as in rodents with T1D [4]. After ICV infusion of an MC4R antagonist, chronic leptin administration failed to reduce plasma insulin and glucose concentrations in non-diabetic rats or to reduce blood glucose in rats with T1D [19]. In addition, mice with MC4R deficiency were completely unresponsive to leptin’s actions to improve insulin sensitivity even when they were pair fed and prevented from becoming obese, indicating that MC4R deficiency, not obesity-induced leptin resistance, abolished the effects of leptin on glucose regulation [4,71]. Also, chronic ICV leptin infusion failed to lower blood glucose in MC4R knock out rats that were severely diabetic due to STZ treatment (Figure 5) [61]. As discussed previously, restoration of LepRs in POMC neurons also reverses hyperglycemia and glucose intolerance in db/db mice. Thus, functional MC4Rs as well as LepRs on POMC neurons are essential for leptin’s chronic antidiabetic actions.

#### Activation of SHP2MAPK signaling in POMC neurons contributes to leptin’s antidiabetic effects

Of leptin’s three main signaling pathways in POMC neurons, stimulation of SHP2–MAPK appears to be especially important in mediating the effects of leptin on regulation of blood glucose levels and BP. Selective deletion of SHP2 in forebrain neurons or specifically in POMC neurons abolished leptin’s ability to reduce blood glucose and insulin levels, suggesting that SHP2–MAPK signaling is critically important for leptin’s chronic antidiabetic effects [49]. Neuronal deletion of IRS2 is associated with modest glucose intolerance and hyperinsulinemia [48], and these effects can be recapitulated by IRS2 deletion specifically in neurons that express LepRs [132]. However, CNS deletion of IRS2 did not impair leptin’s antidiabetic effects in mice with T1D [48]. Deletion of STAT3 in POMC neurons led to moderate increases in body weight but caused no major changes in fasting plasma insulin or glucose concentrations, and did not markedly alter the plasma insulin and glucose responses to chronic ICV infusion of leptin [133]. Thus, although all three of the LepR signaling pathways may contribute to leptin’s CNS-mediated antidiabetic actions in certain conditions, the SHP2–MAPK pathway in POMC neurons appears to be most important.

#### Stimulation of MC4R is necessary but not sufficient to fully recapitulate the chronic antidiabetic effects of leptin

Despite being required for leptin’s chronic antidiabetic actions, activation of MC4R with agonists infused directly into the cerebral ventricles cannot fully recapitulate leptin’s antidiabetic effects in insulin-deficient diabetic rats or mice [4]. Since leptin also inhibits activity of AgRP/NPY neurons that may counteract some of the metabolic effects of activating POMC neurons and MC4R, we tested whether chronic MC4R activation could recapitulate the chronic antidiabetic actions of leptin in NPY-deficient mice with T1D. However, the chronic glucose lowering effects of CNS MC4R activation were not enhanced in NPY-deficient diabetic mice [134]. Thus, activation of the CNS melanocortin pathway may be required for leptin’s powerful antidiabetic actions in some conditions, such as in T1D, but effects of leptin on other neuronal populations also play an important role in other circumstances. For example, leptin’s inhibitory effect on AgRP/NPY neurons, by reducing AgRP, may interact synergistically with activation of POMC neurons to mediate the antidiabetic effects of leptin [135,136]. Although inhibition of AgRP neurons also appears to be critical for the glucose-lowering actions of leptin, this action requires activation of the CNS melanocortin system rather than NPY.

### How does the brain communicate with peripheral tissues to regulate blood glucose during chronic activation of the leptin–melanocortin pathway? Evidence for a CNS-derived circulating factor

Although activation of the CNS leptin–MC4R pathway stimulates the autonomic nervous system and the pituitary–adrenal axis, these actions cannot explain the potent antidiabetic effects of leptin and MC4R activation of the CNS. To determine whether activation of the CNS leptin–melanocortin system may stimulate release of a circulating antidiabetic factor that acts on peripheral tissues, we used a parabiosis protocol in rats with insulin-deficient T1D [137]. Inbred rats with STZ-induced T1D were conjoined by skin-to-skin anastomosis and one of the paired rats received ICV infusion of leptin for 7 days at a dose that does not increase plasma leptin levels while the other rat received ICV infusion of saline vehicle. Chronic ICV leptin infusion restored normoglycemia in leptin-infused rats while reducing blood glucose by ∼27% in conjoined vehicle-infused rats. Reductions in blood glucose were associated with decreased hepatic gluconeogenesis and increased glucose transporter 4 expression in skeletal muscle, the main regulator of tissue glucose uptake.

These findings indicate that brain-mediated antidiabetic effects of leptin–melanocortin system activation are at least partly mediated by release into the systemic circulation of a factor(s) that enhances glucose uptake in peripheral tissues while reducing gluconeogenesis by the liver. However, the identity of the circulating factor that regulates glucose independently of insulin, the autonomic nervous system, and the pituitary–adrenal axis is still unclear and remains an important area for further investigation.

The importance of these brain-mediated effects of activating the leptin–CNS melanocortin system can be demonstrated in non-diabetic animals but are most apparent in T1D. In uncontrolled T1D with severe hyperglycemia, small amounts of leptin infused directly into the cerebrospinal fluid can completely reverse hyperglycemia and diabetic ketoacidosis [138], the most severe effects of insulin deficiency, as well as other cardiorenal consequences such as polyuria, polydipsia, and impaired baroreflexes [4,138,139]. Although the brain circuits, neuronal signaling pathways, and mechanisms by which the brain communicates with peripheral tissues to control blood glucose levels have not been fully elucidated, the CNS melanocortin system plays an essential role in mediating the powerful antidiabetic actions of leptin.

## Cardiorenal protective actions of activating the CNS leptin–melanocortin pathway

Despite its important antidiabetic effects, leptin has also been suggested to cause tissue inflammation and target organ injury, independently of obesity and increased BP. For example, *in vitro* studies suggest that pharmacological levels of leptin may induce vascular smooth muscle proliferation and hypertrophy, oxidative stress in endothelial cells, and reduced contractility as well as hypertrophy of cardiomyocytes [140,141]. Multiple epidemiological studies in humans have also shown that hyperleptinemia is associated with increased risk for cardiovascular and kidney diseases [142–144]. However, these associations are usually confounded by co-dependent risk factors such as obesity and hypertension.

In contrast, several studies have demonstrated that hyperleptinemia and activation of the CNS melanocortin system exert metabolic actions that are highly beneficial for the heart, kidneys, liver and other organs. For example, leptin deficient ob/ob mice display multiple abnormalities of cardiac function including impaired contractile function of ventricular myocytes, reduced fatty acid oxidation, accumulation of cardiac lipids, and cardiac hypertrophy [145–147]. Leptin replacement in ob/ob mice improves cardiac function and reverses cardiac hypertrophy independent of weight loss [146]. Also, cardiomyocyte-specific LepR deletion in lean mice causes cardiac dysfunction [148] whereas rescue of LepRs in cardiomyocytes of obese db/db mice attenuates cardiac lipotoxicity and improves overall heart function [149].

Chronic leptin administration also attenuates cardiac hypertrophy and improves diastolic function in people with generalized lipodystrophy [150]. Other organs, including the kidneys and liver, also benefit from leptin replacement in people with leptin deficiency [151,152]. For example, recombinant leptin therapy reverses severe fatty liver disease in people with lipodystrophy even before significant weight loss and reductions in overall adiposity [152].

Although some of leptin’s cardiorenal and hepatic protective actions may be due to systemic metabolic effects (e.g. reduced visceral obesity and improved glucose regulation) or to direct effects on tissue metabolism, activation of CNS pathways also plays a major role. Multiple studies have shown, for example, that selective stimulation of CNS LepRs or MC4Rs improves mitochondrial function and utilization of glucose and fatty acids in various peripheral tissues [4,45,145,153]. These brain-mediated effects of the leptin–CNS melanocortin system may provide an important therapeutic target for protecting the heart, kidneys and other organs from acute ischemic injury as well as chronic injury due to excessive accumulation of lipids and subsequent ‘lipotoxicity’ [153].

### Activation of brain leptin–melanocortin pathway improves cardiac function after ischemic injury

**Figure 6:**
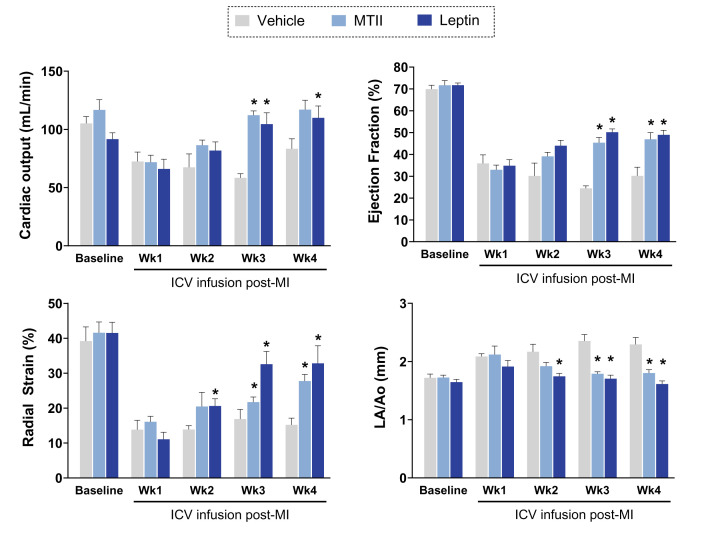
Chronic ICV infusion of leptin or an MC4R agonist improves cardiac function after myocardial infarction ICV Infusion of leptin or melanotan II (MTII), an MC4R antagonist, attenuated cardiac dysfunction in rats after MI. LA/Ao, left atrium/aorta diameter ratio, an index of left atrial enlargement. (Redrawn from data in [154]).

Gava et al. [154] showed in rats that pharmacological activation of the brain leptin–MC4R axis markedly improved cardiac function and attenuated adverse cardiac remodeling after severe myocardial infarction (MI) induced by permanent ligation of the left descending coronary artery (Figure 6). They reported that ICV leptin infusion for 28 days, beginning about 15–20 minutes after MI at rates that have no significant effect on plasma leptin concentrations, nearly normalized left ventricular (LV) ejection fraction, cardiac output, and left atrium/aorta diameter ratio and markedly improved cardiomyocyte strain in non-infarcted regions of the LV. The CNS actions of leptin also attenuated cardiac hypertrophy, cardiomyocyte diameter, and cardiac collagen deposition in the non-infarcted regions of the LV 4 weeks after MI. Although ICV leptin infusion reduced food intake, pair feeding studies showed that leptin’s cardioprotective effects after MI could not be explained by decreased food intake and weight loss [154].

Chronic ICV infusion of MTII, a MC4R agonist, mimicked the effects of leptin to improve cardiac function after MI, whereas genetic deficiency of MC4R completely abolished leptin’s cardioprotective actions. These findings indicate that stimulation of the CNS melanocortin system is essential for leptin’s impressive effects to improve cardiac function after MI [154].

Omoto et al. [155] also observed in rats with ischemia/reperfusion (I/R) injury, caused by occlusion of the left descending coronary artery followed by reperfusion, that ICV leptin infusion greatly improved cardiac function as assessed by echocardiography, LV pressures (maximal rate of LV pressure increase and decrease), and exercise capacity. The CNS-mediated cardiac protection by ICV leptin infusion was equally effective in males and females, and occurred independently of leptin’s anorexic effects [155]. Moreover, these cardioprotective effects did not depend on cardiac sympathetic innervation since bilateral surgical removal of the cervical ganglia did not diminish leptin’s beneficial effects on heart function [155]. Although we did not directly assess the potential role of the parasympathetic system in mediating leptin’s beneficial effects on the heart after ischemic injury, we previously showed that ICV leptin infusion for 7 days restored cardiac vagal tone and normalized baroreflex sensitivity in insulin-deficient rats [139].

#### Metabolic mechanisms for cardioprotection after activation of the brain leptin–melanocortin pathway

Improvements in cardiac function elicited by activating the brain LepRs or MC4Rs after MI were associated with improvements in mitochondrial function and cardiac metabolism in the non-infarcted regions of the heart rather than reductions in the infarction size [154,155]. Although ICV infusion of leptin or an MC4R agonist elicited similar cardioprotective effects after MI, leptin mainly increased myocardial glucose oxidation whereas MC4R activation primarily increased fatty acid oxidation [154]. Leptin and MC4R activation both increased cardiac adenosine monophosphate-activated protein kinase.

The finding of increased myocardial glucose oxidation with ICV leptin infusion or increased fatty acid oxidation with ICV MTII infusion suggests that increased overall mitochondrial oxidative metabolism may be critical for protecting the heart after MI, rather than a specific increase in glucose or fatty acid oxidation [156]. However, it is also possible that increased mitochondrial oxidative metabolism may occur secondary to other factors that improve function of the non-infarcted cardiac muscle, rather than mediating the beneficial effects of leptin and MTII on the heart. Further studies are needed to test these alternate explanations.

#### How does the brain communicate with the heart during activation of the CNS leptin–melanocortin system?

Improvements of overall cardiac function induced by activating the CNS leptin–melanocortin system after MI or I/R injury develop slowly, requiring 2–4 weeks before they can be detected by echocardiography. Although the explanation for this slowly developing response is uncertain, the time lag could be related to the following: (1) slowly developing changes in the brain (e.g. plasticity of neuronal structure or function, or release by the brain of a slowly acting circulating factor that mediates cardioprotection); (2) slowly developing changes in the heart (e.g. structural and/or metabolic); (3) activation of another tissue that communicates with the heart via release of factors (e.g. cytokines, extracellular vesicles (EVs)) that require an extended period of time to exert their protective actions on the heart.

**Figure 7:**
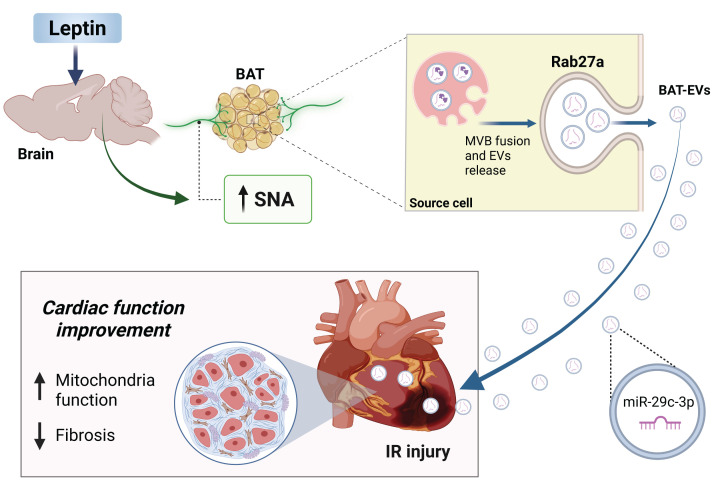
Leptin activates brain-BAT-heart crosstalk to promote cardioprotection after myocardial infarction Chronic infusion of leptin (ICV) may improve cardiac function after I/R injury by stimulating SNA, which activates BAT to release EVs containing cargoes (e.g. miR-29c-3p) that are transported to the heart and attenuate cardiac mitochondrial dysfunction and fibrosis. (Based on data in [157]).

As discussed previously, the cardioprotective effects of activating CNS LepRs or MC4Rs after MI or I/R injury do not appear to be mediated by cardiac sympathetic nerves. However, the SNS may communicate with another peripheral organ/tissue that releases a cardioprotective factor, as mentioned previously. Recent studies by Omoto et al. [157] indicate that chronic ICV leptin infusion stimulates BAT, via the sympathetic nerves, to release EVs that exert cardioprotective effects after I/R injury (Figure 7). Surgical ablation of BAT or sympathetic denervation of BAT greatly diminished the cardioprotective effects of chronic ICV infusion of leptin after I/R injury. Knockdown of BAT Rab27a, a protein required for release of EVs, also reduced the cardioprotective effects of CNS LepR activation. MicroRNA (miR)-29c-3p was identified as a potential cargo of leptin-stimulated BAT-derived EVs that could play a role in attenuating cardiac fibrosis after I/R injury in leptin-treated rats. Overall, these studies provide strong evidence in rodents for brain-BAT-heart crosstalk during activation of CNS LepRs, and that this crosstalk contributes to improved recovery of cardiac function after I/R injury.

Additional studies are needed to determine whether the same factors released by BAT during CNS LepR activation also mediate the cardioprotection elicited by MC4R activation after MI and I/R injury. Importantly, studies will be needed to assess the potential role of cardioprotective factors released from BAT in humans. Although BAT was previously thought to be physiologically relevant only in infants, more recent studies have provided evidence that BAT-like (beige) depots are present and active in adult humans, and may play a role in cardiometabolic health [158,159].

### Activation of brain leptin–melanocortin pathway attenuates ischemic/reperfusion injury of the kidneys

Chronic ICV infusion of leptin also attenuated reductions in glomerular filtration rate (GFR) and acute kidney injury (AKI) in male rats subjected to 30 minutes of complete unilateral renal ischemia followed by reperfusion [160]. The renal protective effects of leptin were independent of changes in food intake and were associated with reductions in several biomarkers of kidney injury, including renal injury scores in the cortex and medulla, decreased excretion of albumin, and reductions in kidney injury molecule-1 and neutrophil gelatinase lipocalin. Thus, leptin’s CNS actions are capable of attenuating kidney as well as cardiac dysfunction after ischemic insults.

Although the cardiac metabolic and protective effects of leptin after ischemia require activation of the brain melanocortin system, the role of POMC neuron activation and stimulation of MC4R in mediating the renal protective effects of leptin after ischemia have not, to our knowledge, been fully assessed. Preliminary evidence [160] suggests that activation of the CNS melanocortin system may play a similar role as in the heart. However, the factors that link stimulation of CNS LepRs and MC4Rs with renal protection after AKI remain to be determined.

An important difference in the renal and cardiac protective actions of activating CNS LepRs is that the functional effects in the kidneys (e.g. improvement of GFR) occur more rapidly (e.g. within 24–48 hours) compared to the heart where significant improvement of function (assessed by echocardiography) required at least 2–3 weeks. The reasons for these differences in the time course for protection after ischemic injury are unclear and warrant further investigation.

## Summary, therapeutic implications for cardiorenal injury, and perspectives

Adipocyte-derived leptin and one of its key CNS mediators, the melanocortin system, have proved to be critical regulators not only of energy balance but also cardiometabolic function. Results from preclinical experiments suggest that activation of CNS LepRs and MC4Rs may provide a potential therapeutic strategy for treating metabolic disorders and for attenuating ischemic injury to the heart, kidneys, and other organs such as the brain [153,160,161]. However, additional studies are needed to establish the mechanisms for inter-organ crosstalk of the brain with peripheral organs and tissues (e.g. BAT) that may contribute to this protection. Further studies are also needed to assess whether continued activation of the CNS leptin–melanocortin system is required to sustain improved organ function after ischemic injury, or whether even temporary activation of this system during a critical period of energy deficit exerts beneficial effects that ultimately lead to long lasting protection of target organs.

Most important, however, is whether these promising preclinical studies will translate to target organ protection in humans. Thus far, studies supporting the possibility that selective activation of CNS LepRs and MC4Rs improves cardiac and kidney function after MI or I/R injury have been conducted in rodents. Since it may be impractical to infuse drugs directly into the brain for therapy of cardiorenal and metabolic diseases in humans, pharmacological agents must readily cross the BBB to be effective in stimulating CNS pathways. Fortunately, recombinant leptin and an FDA-approved MC4R agonist (setmelanotide) are available and cross the BBB, although obesity may attenuate leptin transport into the brain. Most people with obesity already have high levels of leptin and appear to be resistant to its CNS-mediated metabolic effects. This ‘leptin resistance’ likely has important therapeutic implications since many people who suffer MI or I/R injury are obese.

Administration of MC4R agonists may be a more feasible therapeutic strategy than leptin therapy since leptin exerts its protection of ischemic organs mainly by stimulating POMC neurons, ultimately leading to activation of MC4Rs. Previous studies suggest that obesity does not cause resistance to the cardiovascular and metabolic effects of MC4R activation [162]. In fact, obesity enhances rather than reduces responsiveness to MC4R agonists; this enhancement may be secondary to leptin resistance in POMC neurons and upregulation of MC4Rs in downstream neurons [163]. Importantly, MC4R agonists (e.g. setmelanotide) that cross the blood-brain barrier have already been developed and are being used to treat people with rare forms of genetic obesity associated with deficiencies of the LepR or the melanocortin pathway (e.g. POMC deficiency and Bardet-Biedl syndrome) [164].

## Open Access

Open access for this article was enabled by the participation of the University of Mississippi in an all-inclusive Read & Publish agreement with Portland Press and the Biochemical Society.

## Abbreviations

α-MSH: α-melanocyte stimulating hormone
AgRP: agouti-related peptide
AKI: acute kidney injury
ARC: arcuate nucleus
BBB: blood brain barrier
BAT: brown adipose tissue
BDNF: brain-derived neurotrophic factor
BP: blood pressure
CART: cocaine- and amphetamine-regulated transcript
CNS: central nervous system
CYP11B2: aldosterone synthase
DMH: dorsomedial hypothalamus
DMV: dorsal motor nucleus of the vagus
EV: extracellular vesicle
GFR: glomerular filtration rate
HPA: hypothalamic–pituitary–adrenal
HR: heart rate
I/R: ischemia/reperfusion
ICV: intracerebroventricular
IML: intermediolateral nucleus
IRS2: Insulin receptor substrate 2
JAK2: janus tyrosine kinase 2
LepR: leptin receptor
LHA: lateral hypothalamic area
LV: left ventricle
MAPK: mitogen-activated protein kinase
MC3R: melanocortin 3 receptor
MC4R: melanocortin 4 receptor
MI: myocardial infarction
miR: microRNA
MR: mineralocorticoid receptor
MRAP2: melanocortin receptor accessory protein 2
MTII: melanotan II
NO: nitric oxide
NPY: neuropeptide Y
NTS: nucleus tractus solitarius
POMC: proopiomelanocortin
PRA: plasma renin activity
PVN: paraventricular nucleus
RAAS: renin–angiotensin–aldosterone system
RVLM: rostral ventrolateral medulla
SHP2: Src homology-2 tyrosine phosphatase
SHR: spontaneously hypertensive rats
SNA: sympathetic nerve activity
SNS: sympathetic nervous system
STAT3: signal transducers and activators of transcription 3
STZ: streptozotocin
T1D: type 1 diabetes

## References

[1] Friedman J.M. and Halaas J.L. (1998) Leptin and the regulation of body weight in mammals. Nature 395, 763–770 10.1038/27376 9796811

[2] Friedman J.M. (2019) Leptin and the endocrine control of energy balance. Nat. Metab. 1, 754–764 10.1038/s42255-019-0095-y 32694767

[3] Ahima R.S. and Flier J.S. (2026) Leptin: 30 years later. Annu. Rev. Physiol. 88, 229–250 10.1146/annurev-physiol-042324-100259 41043250

[4] da Silva A.A., do Carmo J.M. and Hall J.E. (2020) CNS regulation of glucose homeostasis: role of the leptin–melanocortin system. Curr. Diab. Rep. 20, 29 10.1007/s11892-020-01311-1 32451760

[5] Morton G.J. and Schwartz M.W. (2011) Leptin and the central nervous system control of glucose metabolism. Physiol. Rev. 91, 389–411 10.1152/physrev.00007.2010 21527729 PMC3379883

[6] Shek E.W., Brands M.W. and Hall J.E. (1998) Chronic leptin infusion increases arterial pressure. Hypertension 31, 409–414 10.1161/01.HYP.31.1.409 9453337

[7] Haynes W.G., Morgan D.A., Walsh S.A., Sivitz W.I. and Mark A.L. (1998) Cardiovascular consequences of obesity: role of leptin. Clin. Exp. Pharmacol. Physiol. 25, 65–69 10.1111/j.1440-1681.1998.tb02147.x 9493562

[8] Hall J.E., da Silva A.A., do Carmo J.M., Dubinion J., Hamza S., Munusamy S. et al. (2010) Obesity-induced hypertension: role of sympathetic nervous system, leptin, and melanocortins. J. Biol. Chem. 285, 17271–17276 10.1074/jbc.R110.113175 20348094 PMC2878489

[9] do Carmo J.M., da Silva A.A., Dubinion J., Sessums P.O., Ebaady S.H., Wang Z. et al. (2013) Control of metabolic and cardiovascular function by the leptin–brain melanocortin pathway. IUBMB Life 65, 692–698 10.1002/iub.1187 23847053 PMC4077663

[10] Nijenhuis W.A., Oosterom J. and Adan R.A. (2001) AgRP(83–132) acts as an inverse agonist on the human-melanocortin-4 receptor. Mol. Endocrinol. 15, 164–171 11145747 10.1210/mend.15.1.0578

[11] Breit A., Wolff K., Kalwa H., Jarry H., Buch T. and Gudermann T. (2006) The natural inverse agonist agouti-related protein induces arrestin-mediated endocytosis of melanocortin-3 and -4 receptors. J. Biol. Chem. 281, 37447–37456 10.1074/jbc.M605982200 17041250

[12] Farooqi S. and O’Rahilly S. (2006) Genetics of obesity in humans. Endocr. Rev. 27, 710–718 10.1210/er.2006-0040 17122358

[13] Mountjoy K.G. (2015) Pro-opiomelanocortin (POMC) neurones, POMC-derived peptides, melanocortin receptors and obesity: how understanding of this system has changed over the last decade. J. Neuroendocrinol. 27, 406–418 10.1111/jne.12285 25872650

[14] Sweeney P., Gimenez L.E., Hernandez C.C. and Cone R.D. (2023) Targeting the central melanocortin system for the treatment of metabolic disorders. Nat. Rev. Endocrinol. 19, 507–519 10.1038/s41574-023-00855-y 37365323

[15] Kuo J.J., Silva A.A. and Hall J.E. (2003) Hypothalamic melanocortin receptors and chronic regulation of arterial pressure and renal function. Hypertension 41, 768–774 10.1161/01.HYP.0000048194.97428.1A 12623994

[16] da Silva A.A., do Carmo J.M., Wang Z. and Hall J.E. (2019) Melanocortin-4 receptors and sympathetic nervous system activation in hypertension. Curr. Hypertens. Rep. 21, 46 10.1007/s11906-019-0951-x 31028563 PMC7213056

[17] do Carmo J.M., da Silva A.A., Wang Z., Fang T., Aberdein N., Perez de Lara C.E. et al. (2017) Role of the brain melanocortins in blood pressure regulation. Biochim. Biophys. Acta 1863, 2508–2514 10.1016/j.bbadis.2017.03.003 PMC5587353 28274841

[18] Hall J.E., do Carmo J.M., da Silva A.A., Wang Z. and Hall M.E. (2019) Obesity, kidney dysfunction and hypertension: mechanistic links. Nat. Rev. Nephrol. 15, 367–385 10.1038/s41581-019-0145-4 31015582 PMC7278043

[19] da Silva A.A., Kuo J.J. and Hall J.E. (2004) Role of hypothalamic melanocortin 3/4-receptors in mediating chronic cardiovascular, renal, and metabolic actions of leptin. Hypertension 43, 1312–1317 10.1161/01.HYP.0000128421.23499.b9 15123576

[20] Rahmouni K., Haynes W.G., Morgan D.A. and Mark A.L. (2003) Role of melanocortin-4 receptors in mediating renal sympathoactivation to leptin and insulin. J. Neurosci. 23, 5998–6004 10.1523/JNEUROSCI.23-14-05998.2003 12853417 PMC6740337

[21] Haynes W.G., Morgan D.A., Walsh S.A., Mark A.L. and Sivitz W.I. (1997) Receptor-mediated regional sympathetic nerve activation by leptin. J. Clin. Invest. 100, 270–278 10.1172/JCI119532 9218503 PMC508189

[22] Machleidt F., Simon P., Krapalis A.F., Hallschmid M., Lehnert H. and Sayk F. (2013) Experimental hyperleptinemia acutely increases vasoconstrictory sympathetic nerve activity in healthy humans. J. Clin. Endocrinol. Metab. 98, E491–E496 10.1210/jc.2012-3009 23393176

[23] Snitker S., Pratley R.E., Nicolson M., Tataranni P.A. and Ravussin E. (1997) Relationship between muscle sympathetic nerve activity and plasma leptin concentration. Obes. Res. 5, 338–340 10.1002/j.1550-8528.1997.tb00561.x 9285841

[24] Ozata M., Ozdemir I.C. and Licinio J. (1999) Human leptin deficiency caused by a missense mutation: multiple endocrine defects, decreased sympathetic tone, and immune system dysfunction indicate new targets for leptin action, greater central than peripheral resistance to the effects of leptin, and spontaneous correction of leptin-mediated defects. J. Clin. Endocrinol. Metab. 84, 3686–3695 10.1210/jcem.84.10.5999 10523015

[25] Mark A.L., Shaffer R.A., Correia M.L., Morgan D.A., Sigmund C.D. and Haynes W.G. (1999) Contrasting blood pressure effects of obesity in leptin-deficient ob/ob mice and agouti yellow obese mice. J. Hypertens. 17, 1949–1953 10.1097/00004872-199917121-00026 10703894

[26] Shi Z. and Brooks V.L. (2015) Leptin differentially increases sympathetic nerve activity and its baroreflex regulation in female rats: role of oestrogen. J. Physiol. 593, 1633–1647 10.1113/jphysiol.2014.284638 25398524 PMC4386963

[27] da Silva A.A., Pinkerton M.A., Spradley F.T., Palei A.C., Hall J.E. and do Carmo J.M. (2021) Chronic CNS-mediated cardiometabolic actions of leptin: potential role of sex differences. Am. J. Physiol. Regul. Integr. Comp. Physiol. 320, R173–R181 10.1152/ajpregu.00027.2020 33206555 PMC7948126

[28] Hall J.E., do Carmo J.M., da Silva A.A., Wang Z. and Hall M.E. (2015) Obesity-induced hypertension: interaction of neurohumoral and renal mechanisms. Circ. Res. 116, 991–1006 10.1161/CIRCRESAHA.116.305697 25767285 PMC4363087

[29] Aizawa-Abe M., Ogawa Y., Masuzaki H., Ebihara K., Satoh N., Iwai H. et al. (2000) Pathophysiological role of leptin in obesity-related hypertension. J. Clin. Invest. 105, 1243–1252 10.1172/JCI8341 10791999 PMC315441

[30] Carlyle M., Jones O.B., Kuo J.J. and Hall J.E. (2002) Chronic cardiovascular and renal actions of leptin: role of adrenergic activity. Hypertension 39, 496–501 10.1161/hy0202.104398 11882597

[31] Kuo J.J., Jones O.B. and Hall J.E. (2001) Inhibition of NO synthesis enhances chronic cardiovascular and renal actions of leptin. Hypertension 37, 670–676 10.1161/01.HYP.37.2.670 11230354

[32] Mark A.L. (2013) Selective leptin resistance revisited. Am. J. Physiol. Regul. Integr. Comp. Physiol. 305, R566–R581 10.1152/ajpregu.00180.2013 23883674 PMC3763044

[33] Heymsfield S.B., Greenberg A.S., Fujioka K., Dixon R.M., Kushner R., Hunt T. et al. (1999) Recombinant leptin for weight loss in obese and lean adults: a randomized, controlled, dose-escalation trial. JAMA 282, 1568–1575 10.1001/jama.282.16.1568 10546697

[34] Brown R.J., Meehan C.A. and Gorden P. (2015) Leptin does not mediate hypertension associated with human obesity. Cell 162, 465–466 10.1016/j.cell.2015.07.007 26232214 PMC7262432

[35] Simonds S.E., Pryor J.T., Ravussin E., Greenway F.L., Dileone R., Allen A.M. et al. (2014) Leptin mediates the increase in blood pressure associated with obesity. Cell 159, 1404–1416 10.1016/j.cell.2014.10.058 25480301 PMC4259491

[36] Lim K., Burke S.L. and Head G.A. (2013) Obesity-related hypertension and the role of insulin and leptin in high-fat-fed rabbits. Hypertension 61, 628–634 10.1161/HYPERTENSIONAHA.111.00705 23339171

[37] von Schnurbein J., Manzoor J., Brandt S., Denzer F., Kohlsdorf K., Fischer-Posovszky P. et al. (2019) Leptin is not essential for obesity-associated hypertension. Obes Facts 12, 460–475 10.1159/000501319 31357197 PMC6758712

[38] Couillard C., Mauriege P., Prud’homme D., Nadeau A., Tremblay A., Bouchard C. et al. (1997) Plasma leptin concentrations: gender differences and associations with metabolic risk factors for cardiovascular disease. Diabetologia 40, 1178–1184 10.1007/s001250050804 9349599

[39] Narkiewicz K., Phillips B.G., Kato M., Hering D., Bieniaszewski L. and Somers V.K. (2005) Gender-selective interaction between aging, blood pressure, and sympathetic nerve activity. Hypertension 45, 522–525 10.1161/01.HYP.0000160318.46725.46 15767469

[40] Yanes L.L. and Reckelhoff J.F. (2011) Postmenopausal hypertension. Am. J. Hypertens. 24, 740–749 10.1038/ajh.2011.71 21509049 PMC3820162

[41] Faria A.N., Ribeiro Filho F.F., Gouveia Ferreira S.R. and Zanella M.T. (2002) Impact of visceral fat on blood pressure and insulin sensitivity in hypertensive obese women. Obes. Res. 10, 1203–1206 10.1038/oby.2002.164 12490663

[42] Hall J.E., Mouton A.J., da Silva A.A., Omoto A.C.M., Wang Z., Li X. et al. (2021) Obesity, kidney dysfunction, and inflammation: interactions in hypertension. Cardiovasc. Res. 117, 1859–1876 10.1093/cvr/cvaa336 33258945 PMC8262632

[43] Foster M.C., Hwang S.J., Porter S.A., Massaro J.M., Hoffmann U. and Fox C.S. (2011) Fatty kidney, hypertension, and chronic kidney disease: the Framingham Heart Study. Hypertension 58, 784–790 10.1161/HYPERTENSIONAHA.111.175315 21931075 PMC3204377

[44] Gu J.W., Wang J., Stockton A., Lokitz B., Henegar L. and Hall J.E. (2004) Cytokine gene expression profiles in kidney medulla and cortex of obese hypertensive dogs. Kidney Int. 66, 713–721 10.1111/j.1523-1755.2004.00793.x 15253726

[45] Zhao Y., Laule C. and Rahmouni K. (2025) Central leptin pathways in metabolic homeostasis. Clin. Sci. (Lond.) 139, 1451–1468 10.1042/CS20257748 41251447 PMC12751087

[46] Harlan S.M. and Rahmouni K. (2013) Neuroanatomical determinants of the sympathetic nerve responses evoked by leptin. Clin. Auton. Res. 23, 1–7 10.1007/s10286-012-0168-4 22714900 PMC3496820

[47] Pan W.W. and Myers M.G. Jr. (2018) Leptin and the maintenance of elevated body weight. Nat. Rev. Neurosci. 19, 95–105 10.1038/nrn.2017.168 29321684

[48] do Carmo J.M., da Silva A.A., Wang Z., Freeman N.J., Alsheik A.J., Adi A. et al. (2016) Regulation of blood pressure, appetite, and glucose by leptin after inactivation of insulin receptor substrate 2 signaling in the entire brain or in proopiomelanocortin neurons. Hypertension 67, 378–386 10.1161/HYPERTENSIONAHA.115.06153 26628674 PMC4763950

[49] do Carmo J.M., da Silva A.A., Sessums P.O., Ebaady S.H., Pace B.R., Rushing J.S. et al. (2014) Role of Shp2 in forebrain neurons in regulating metabolic and cardiovascular functions and responses to leptin. Int. J. Obes. (Lond.) 38, 775–783 10.1038/ijo.2013.177 24030516 PMC3954949

[50] do Carmo J.M., da Silva A.A., Ebaady S.E., Sessums P.O., Abraham R.S., Elmquist J.K. et al. (2014) Shp2 signaling in POMC neurons is important for leptin’s actions on blood pressure, energy balance, and glucose regulation. Am. J. Physiol. Regul. Integr. Comp. Physiol. 307, R1438–R1447 10.1152/ajpregu.00131.2014 25339680 PMC4269667

[51] Mountjoy K.G. (2010) Distribution and function of melanocortin receptors within the brain. Adv. Exp. Med. Biol. 681, 29–48 10.1007/978-1-4419-6354-3_3 21222258

[52] Toda C., Santoro A., Kim J.D. and Diano S. (2017) POMC neurons: from birth to death. Annu. Rev. Physiol. 79, 209–236 10.1146/annurev-physiol-022516-034110 28192062 PMC5669621

[53] Ruggiero-Ruff R.E. and Coss D. (2025) Neuroendocrinology and the genetics of obesity. Endocrinology 166, bqaf121 10.1210/endocr/bqaf121 40690308 PMC12342173

[54] Farooqi I.S., Yeo G.S., Keogh J.M., Aminian S., Jebb S.A., Butler G. et al. (2000) Dominant and recessive inheritance of morbid obesity associated with melanocortin 4 receptor deficiency. J. Clin. Invest. 106, 271–279 10.1172/JCI9397 10903343 PMC314308

[55] Vaisse C., Clement K., Durand E., Hercberg S., Guy-Grand B. and Froguel P. (2000) Melanocortin-4 receptor mutations are a frequent and heterogeneous cause of morbid obesity. J. Clin. Invest. 106, 253–262 10.1172/JCI9238 10903341 PMC314306

[56] Farooqi I.S. and O’Rahilly S. (2008) Mutations in ligands and receptors of the leptin–melanocortin pathway that lead to obesity. Nat Clin Pract Endocrinol Metab. 4, 569–577 10.1038/ncpendmet0966 18779842

[57] Wade K.H., Lam B.Y.H., Melvin A., Pan W., Corbin L.J., Hughes D.A. et al. (2021) Loss-of-function mutations in the melanocortin 4 receptor in a UK birth cohort. Nat. Med. 27, 1088–1096 10.1038/s41591-021-01349-y 34045736 PMC7611835

[58] do Carmo J.M., da Silva A.A., Gava F.N., Moak S.P., Dai X. and Hall J.E. (2019) Impact of leptin deficiency compared with neuronal-specific leptin receptor deletion on cardiometabolic regulation. Am. J. Physiol. Regul. Integr. Comp. Physiol. 317, R552–R562 10.1152/ajpregu.00077.2019 31411897 PMC6842908

[59] do Carmo J.M., da Silva A.A., Cai Z., Lin S., Dubinion J.H. and Hall J.E. (2011) Control of blood pressure, appetite, and glucose by leptin in mice lacking leptin receptors in proopiomelanocortin neurons. Hypertension 57, 918–926 10.1161/HYPERTENSIONAHA.110.161349 21422382 PMC3092393

[60] do Carmo J.M., da Silva A.A., Rushing J.S., Pace B. and Hall J.E. (2013) Differential control of metabolic and cardiovascular functions by melanocortin-4 receptors in proopiomelanocortin neurons. Am. J. Physiol. Regul. Integr. Comp. Physiol. 305, R359–R368 10.1152/ajpregu.00518.2012 23842677 PMC3833394

[61] da Silva A.A., do Carmo J.M., Freeman J.N., Tallam L.S. and Hall J.E. (2009) A functional melanocortin system may be required for chronic CNS-mediated antidiabetic and cardiovascular actions of leptin. Diabetes 58, 1749–1756 10.2337/db08-1221 19491210 PMC2712780

[62] Haynes W.G., Morgan D.A., Djalali A., Sivitz W.I. and Mark A.L. (1999) Interactions between the melanocortin system and leptin in control of sympathetic nerve traffic. Hypertension 33, 542–547 10.1161/01.HYP.33.1.542 9931162

[63] Kuo J.J., da Silva A.A., Tallam L.S. and Hall J.E. (2004) Role of adrenergic activity in pressor responses to chronic melanocortin receptor activation. Hypertension 43, 370–375 10.1161/01.HYP.0000111836.54204.93 14707160

[64] Greenfield J.R., Miller J.W., Keogh J.M., Henning E., Satterwhite J.H., Cameron G.S. et al. (2009) Modulation of blood pressure by central melanocortinergic pathways. N. Engl. J. Med. 360, 44–52 10.1056/NEJMoa0803085 19092146

[65] Kievit P., Halem H., Marks D.L., Dong J.Z., Glavas M.M., Sinnayah P. et al. (2013) Chronic treatment with a melanocortin-4 receptor agonist causes weight loss, reduces insulin resistance, and improves cardiovascular function in diet-induced obese rhesus macaques. Diabetes 62, 490–497 10.2337/db12-0598 23048186 PMC3554387

[66] do Carmo J.M., Dai X., Aitken N., Larson K.M., Omoto A.C.M., Gulke R.R. et al. (2023) Sex differences in weight gain, blood pressure control, and responses to melanocortin-4 receptor antagonism in offspring from lean and obese parents. Am. J. Physiol. Regul. Integr. Comp. Physiol. 325, R401–R410 10.1152/ajpregu.00106.2023 37519251 PMC10639017

[67] do Carmo J.M., da Silva A.A., Moak S.P., Houghton H.J., Smith A. and Hall J.E. (2016) Regulation of blood pressure, appetite, and glucose by CNS melanocortin system in hyperandrogenemic female SHR. Am. J. Hypertens. 29, 832–840 10.1093/ajh/hpv182 26584577 PMC4901857

[68] da Silva A.A., do Carmo J.M., Kanyicska B., Dubinion J., Brandon E. and Hall J.E. (2008) Endogenous melanocortin system activity contributes to the elevated arterial pressure in spontaneously hypertensive rats. Hypertension 51, 884–890 10.1161/HYPERTENSIONAHA.107.100636 18285617 PMC2803054

[69] Tallam L.S., Kuo J.J., da Silva A.A. and Hall J.E. (2004) Cardiovascular, renal, and metabolic responses to chronic central administration of agouti-related peptide. Hypertension 44, 853–858 10.1161/01.HYP.0000148993.47498.b2 15545513

[70] Tallam L.S., Stec D.E., Willis M.A., da Silva A.A. and Hall J.E. (2005) Melanocortin-4 receptor-deficient mice are not hypertensive or salt-sensitive despite obesity, hyperinsulinemia, and hyperleptinemia. Hypertension 46, 326–332 10.1161/01.HYP.0000175474.99326.bf 16027245

[71] Tallam L.S., da Silva A.A. and Hall J.E. (2006) Melanocortin-4 receptor mediates chronic cardiovascular and metabolic actions of leptin. Hypertension 48, 58–64 10.1161/01.HYP.0000227966.36744.d9 16754792

[72] da Silva A.A., Spradley F.T., Granger J.P., Hall J.E. and do Carmo J.M. (2015) Brain-mediated antidiabetic, anorexic, and cardiovascular actions of leptin require melanocortin-4 receptor signaling. J. Neurophysiol. 113, 2786–2791 10.1152/jn.00911.2014 25717164 PMC4416609

[73] Maranon R., Lima R., Spradley F.T., do Carmo J.M., Zhang H., Smith A.D. et al. (2015) Roles for the sympathetic nervous system, renal nerves, and CNS melanocortin-4 receptor in the elevated blood pressure in hyperandrogenemic female rats. Am. J. Physiol. Regul. Integr. Comp. Physiol. 308, R708–R713 10.1152/ajpregu.00411.2014 25695289 PMC4398855

[74] do Carmo J.M., da Silva A.A., Moak S.P., da Silva F.S., Spradley F.T. and Hall J.E. (2019) Role of melanocortin 4 receptor in hypertension induced by chronic intermittent hypoxia. Acta Physiol. (Oxf.) 225, e13222 10.1111/apha.13222 30466186 PMC6416058

[75] Samuelsson A.S., Mullier A., Maicas N., Oosterhuis N.R., Eun Bae S., Novoselova T.V. et al. (2016) Central role for melanocortin-4 receptors in offspring hypertension arising from maternal obesity. Proc. Natl. Acad. Sci. U.S.A. 113, 12298–12303 10.1073/pnas.1607464113 27791019 PMC5087049

[76] Greenfield J.R. (2011) Melanocortin signalling and the regulation of blood pressure in human obesity. J. Neuroendocrinol. 23, 186–193 10.1111/j.1365-2826.2010.02088.x 21062377

[77] Sayk F., Heutling D., Dodt C., Iwen K.A., Wellhoner J.P., Scherag S. et al. (2010) Sympathetic function in human carriers of melanocortin-4 receptor gene mutations. J. Clin. Endocrinol. Metab. 95, 1998–2002 10.1210/jc.2009-2297 20147580

[78] do Carmo J.M., da Silva A.A., Rushing J.S. and Hall J.E. (2012) Activation of the central melanocortin system contributes to the increased arterial pressure in obese Zucker rats. Am. J. Physiol. Regul. Integr. Comp. Physiol. 302, R561–R567 10.1152/ajpregu.00392.2011 22204957 PMC3311523

[79] Simonds S.E., Pryor J.T., Lam B.Y.H., Dowsett G.K., Mustafa T., Munder A. et al. (2025) The metabolic and cardiovascular effects of amphetamine are partially mediated by the central melanocortin system. Cell. Rep. Med. 6, 101936 10.1016/j.xcrm.2025.101936 39914386 PMC11866487

[80] da Silva A.A., do Carmo J.M., Dubinion J.H., Bassi M., Mokhtarpouriani K., Hamza S.M. et al. (2015) Chronic central nervous system MC3/4R blockade attenuates hypertension induced by nitric oxide synthase inhibition but not by angiotensin II infusion. Hypertension 65, 171–177 10.1161/HYPERTENSIONAHA.114.03999 25287400 PMC4267912

[81] Bassi M., Nakamura N.B., Furuya W.I., Colombari D.S., Menani J.V., do Carmo J.M. et al. (2015) Activation of the brain melanocortin system is required for leptin-induced modulation of chemorespiratory function. Acta Physiol. (Oxf.) 213, 893–901 10.1111/apha.12394 25207799 PMC4362918

[82] Bassi M., Furuya W.I., Zoccal D.B., Menani J.V., Colombari E., Hall J.E. et al. (2015) Control of respiratory and cardiovascular functions by leptin. Life Sci. 125, 25–31 10.1016/j.lfs.2015.01.019 25645056 PMC4355938

[83] Bassi M., Giusti H., Leite C.M., Anselmo-Franci J.A., do Carmo J.M., da Silva A.A. et al. (2012) Central leptin replacement enhances chemorespiratory responses in leptin-deficient mice independent of changes in body weight. Pflugers Arch. 464, 145–153 10.1007/s00424-012-1111-1 22585210 PMC4077668

[84] Polotsky V.Y., Smaldone M.C., Scharf M.T., Li J., Tankersley C.G., Smith P.L. et al. (2004) Impact of interrupted leptin pathways on ventilatory control. J. Appl. Physiol. (1985) 96, 991–998 10.1152/japplphysiol.00926.2003 14578371

[85] Pauza A.G., Thakkar P., Shen X., Felippe I.S.A., Rossmann K., Oya M. et al. (2025) Melanocortin system activates carotid body arterial chemoreceptors in hypertension. Circ. Res. 137, 967–982 10.1161/CIRCRESAHA.125.326394 40874981 PMC12435264

[86] Li P., Cui B.P., Zhang L.L., Sun H.J., Liu T.Y. and Zhu G.Q. (2013) Melanocortin 3/4 receptors in paraventricular nucleus modulate sympathetic outflow and blood pressure. Exp. Physiol. 98, 435–443 10.1113/expphysiol.2012.067256 22872662

[87] Ward K.R., Bardgett J.F., Wolfgang L. and Stocker S.D. (2011) Sympathetic response to insulin is mediated by melanocortin 3/4 receptors in the hypothalamic paraventricular nucleus. Hypertension 57, 435–441 10.1161/HYPERTENSIONAHA.110.160671 21263116 PMC3580160

[88] Sohn J.W., Harris L.E., Berglund E.D., Liu T., Vong L., Lowell B.B. et al. (2013) Melanocortin 4 receptors reciprocally regulate sympathetic and parasympathetic preganglionic neurons. Cell 152, 612–619 10.1016/j.cell.2012.12.022 23374353 PMC3711728

[89] Bariohay B., Roux J., Tardivel C., Trouslard J., Jean A. and Lebrun B. (2009) Brain-derived neurotrophic factor/tropomyosin-related kinase receptor type B signaling is a downstream effector of the brainstem melanocortin system in food intake control. Endocrinology 150, 2646–2653 10.1210/en.2008-1184 19179431

[90] Nicholson J.R., Peter J.C., Lecourt A.C., Barde Y.A. and Hofbauer K.G. (2007) Melanocortin-4 receptor activation stimulates hypothalamic brain-derived neurotrophic factor release to regulate food intake, body temperature and cardiovascular function. J. Neuroendocrinol. 19, 974–982 10.1111/j.1365-2826.2007.01610.x 18001327

[91] Tao Y.X. (2010) The melanocortin-4 receptor: physiology, pharmacology, and pathophysiology. Endocr. Rev. 31, 506–543 10.1210/er.2009-0037 20190196 PMC3365848

[92] Sohail I., Laurin S.A., Kleinau G., Chunilal V., Morton A., Brenlla A. et al. (2025) MRAP2 modifies the signaling and oligomerization state of the melanocortin-4 receptor. Nat. Commun. 16, 8324 10.1038/s41467-025-63988-w 40998819 PMC12462500

[93] Guo D.F., Williams P.A., Olson A., Morgan D.A., Herz H., Resch J. et al. (2025) Loss of melanocortin receptor accessory protein 2 in melanocortin-4 receptor neurons protect from obesity-associated autonomic and cardiovascular dysfunctions. Cardiovasc. Res. 121, 1929–1940 10.1093/cvr/cvaf067 40244925 PMC12551390

[94] Wyatt R.A., Jamaluddin A., Mistry V., Quinn C. and Gorvin C.M. (2025) Obesity-associated MRAP2 variants impair multiple MC4R-mediated signaling pathways. Hum. Mol. Genet. 34, 533–546 10.1093/hmg/ddaf005 39807633 PMC11891872

[95] Metzger P.J., Zhang A., Carlson B.A., Sun H., Cui Z., Li Y. et al. (2024) A human obesity-associated MC4R mutation with defective Gq/11alpha signaling leads to hyperphagia in mice. J. Clin. Invest. 134, e165418 10.1172/JCI165418 38175730 PMC10869179

[96] Li Y.Q., Shrestha Y., Pandey M., Chen M., Kablan A., Gavrilova O. et al. (2016) G(q/11)alpha and G(s)alpha mediate distinct physiological responses to central melanocortins. J. Clin. Invest. 126, 40–49 10.1172/JCI76348 26595811 PMC4701544

[97] Butler A.A., Kesterson R.A., Khong K., Cullen M.J., Pelleymounter M.A., Dekoning J. et al. (2000) A unique metabolic syndrome causes obesity in the melanocortin-3 receptor-deficient mouse. Endocrinology 141, 3518–3521 10.1210/endo.141.9.7791 10965927

[98] Ni X.P., Pearce D., Butler A.A., Cone R.D. and Humphreys M.H. (2003) Genetic disruption of gamma-melanocyte-stimulating hormone signaling leads to salt-sensitive hypertension in the mouse. J. Clin. Invest. 111, 1251–1258 10.1172/JCI200316993 12697744 PMC152936

[99] Humphreys M.H., Ni X.P. and Pearce D. (2011) Cardiovascular effects of melanocortins. Eur. J. Pharmacol. 660, 43–52 10.1016/j.ejphar.2010.10.102 21199648 PMC3086937

[100] Butler A.A., Girardet C., Mavrikaki M., Trevaskis J.L., Macarthur H., Marks D.L. et al. (2017) A life without hunger: the ups (and downs) to modulating melanocortin-3 receptor signaling. Front. Neurosci. 11, 128 10.3389/fnins.2017.00128 28360832 PMC5352694

[101] Seiler J.L., Impastato A.C., Zhang E., Kelley K.J., Bennet T.L., Studnitzer B. et al. (2026) Dual activation of MC3R and MC4R drives weight loss and reduces food intake in obese primates. Nat Commun. 17 4808 10.1038/s41467-026-73372-x 42215442 PMC13221471

[102] Parisien-La Salle S., Tsai C.H., Newman A.J., Chan J.M., Milks J., Adler G. et al. (2026) Unmasking hormonal mechanisms of hypertension in obesity. JACC Basic Transl. Sci. 11, 101526 10.1016/j.jacbts.2026.101526 41864192 PMC13022646

[103] Flack J.M., Buhnerkempe M.G., Lopez A. and Byrd J.B. (2026) Link of obesity with primary aldosteronism: causal or not? Hypertension 83, e26227 10.1161/HYPERTENSIONAHA.126.26227 42017239

[104] Huby A.C., Antonova G., Groenendyk J., Gomez-Sanchez C.E., Bollag W.B., Filosa J.A. et al. (2015) Adipocyte-derived hormone leptin is a direct regulator of aldosterone secretion, which promotes endothelial dysfunction and cardiac fibrosis. Circulation 132, 2134–2145 10.1161/CIRCULATIONAHA.115.018226 26362633

[105] Williams B., MacDonald T.M., Morant S., Webb D.J., Sever P., McInnes G. et al. (2015) Spironolactone versus placebo, bisoprolol, and doxazosin to determine the optimal treatment for drug-resistant hypertension (PATHWAY-2): a randomised, double-blind, crossover trial. Lancet 386, 2059–2068 10.1016/S0140-6736(15)00257-3 26414968 PMC4655321

[106] Farooqi I.S., Keogh J.M., Yeo G.S., Lank E.J., Cheetham T. and O’Rahilly S. (2003) Clinical spectrum of obesity and mutations in the melanocortin 4 receptor gene. N. Engl. J. Med. 348, 1085–1095 10.1056/NEJMoa022050 12646665

[107] Petersen K.F., Oral E.A., Dufour S., Befroy D., Ariyan C., Yu C. et al. (2002) Leptin reverses insulin resistance and hepatic steatosis in patients with severe lipodystrophy. J. Clin. Invest. 109, 1345–1350 10.1172/JCI0215001 12021250 PMC150981

[108] Pelleymounter M.A., Cullen M.J., Baker M.B., Hecht R., Winters D., Boone T. et al. (1995) Effects of the obese gene product on body weight regulation in ob/ob mice. Science 269, 540–543 10.1126/science.7624776 7624776

[109] German J.P., Thaler J.P., Wisse B.E., Oh I.S., Sarruf D.A., Matsen M.E. et al. (2011) Leptin activates a novel CNS mechanism for insulin-independent normalization of severe diabetic hyperglycemia. Endocrinology 152, 394–404 10.1210/en.2010-0890 21159853 PMC3037161

[110] da Silva A.A., Tallam L.S., Liu J. and Hall J.E. (2006) Chronic antidiabetic and cardiovascular actions of leptin: role of CNS and increased adrenergic activity. Am. J. Physiol. Regul. Integr. Comp. Physiol. 291, R1275–R1282 10.1152/ajpregu.00187.2006 16778068

[111] Coppari R. and Bjorbaek C. (2012) Leptin revisited: its mechanism of action and potential for treating diabetes. Nat. Rev. Drug Discov. 11, 692–708 10.1038/nrd3757 22935803 PMC4019022

[112] Munzberg H. and Myers M.G. Jr. (2005) Molecular and anatomical determinants of central leptin resistance. Nat. Neurosci. 8, 566–570 10.1038/nn1454 15856064

[113] Banks W.A. (2021) Leptin and the blood–brain barrier: curiosities and controversies. Compr. Physiol. 11, 2351–2369 10.1002/j.2040-4603.2021.tb00183.x 34558673

[114] Tan B., Hedbacker K., Kelly L., Zhang Z., Moura-Assis A., Luo J.D. et al. (2025) A cellular and molecular basis of leptin resistance. Cell. Metab. 37, 723.e726–741.e726 10.1016/j.cmet.2025.01.001 40043692

[115] Ottaway N., Mahbod P., Rivero B., Norman L.A., Gertler A., D’Alessio D.A. et al. (2015) Diet-induced obese mice retain endogenous leptin action. Cell. Metab. 21, 877–882 10.1016/j.cmet.2015.04.015 25980347 PMC4456263

[116] Dubinion J.H., da Silva A.A. and Hall J.E. (2010) Enhanced blood pressure and appetite responses to chronic central melanocortin-3/4 receptor blockade in dietary-induced obesity. J. Hypertens. 28, 1466–1470 10.1097/HJH.0b013e328339f20e 20442673 PMC2892001

[117] Correia M.L., Haynes W.G., Rahmouni K., Morgan D.A., Sivitz W.I. and Mark A.L. (2002) The concept of selective leptin resistance: evidence from agouti yellow obese mice. Diabetes 51, 439–442 10.2337/diabetes.51.2.439 11812752

[118] Rahmouni K., Morgan D.A., Morgan G.M., Mark A.L. and Haynes W.G. (2005) Role of selective leptin resistance in diet-induced obesity hypertension. Diabetes 54, 2012–2018 10.2337/diabetes.54.7.2012 15983201

[119] Coppari R., Ichinose M., Lee C.E., Pullen A.E., Kenny C.D., McGovern R.A. et al. (2005) The hypothalamic arcuate nucleus: a key site for mediating leptin’s effects on glucose homeostasis and locomotor activity. Cell. Metab. 1, 63–72 10.1016/j.cmet.2004.12.004 16054045

[120] Huo L., Gamber K., Greeley S., Silva J., Huntoon N., Leng X.H. et al. (2009) Leptin-dependent control of glucose balance and locomotor activity by POMC neurons. Cell. Metab. 9, 537–547 10.1016/j.cmet.2009.05.003 19490908 PMC2730605

[121] Laule C. and Rahmouni K. (2025) Leptin and associated neural pathways underlying obesity-induced hypertension. Compr. Physiol. 15, e8 10.1002/cph4.8 40293220 PMC12038170

[122] do Carmo J.M., da Silva A.A., Wang Z., Fang T., Aberdein N., de Lara Rodriguez C.E. et al. (2016) Obesity-induced hypertension: brain signaling pathways. Curr. Hypertens. Rep. 18, 58 10.1007/s11906-016-0658-1 27262997 PMC5448788

[123] Haque M.S., Minokoshi Y., Hamai M., Iwai M., Horiuchi M. and Shimazu T. (1999) Role of the sympathetic nervous system and insulin in enhancing glucose uptake in peripheral tissues after intrahypothalamic injection of leptin in rats. Diabetes 48, 1706–1712 10.2337/diabetes.48.9.1706 10480598

[124] Kamohara S., Burcelin R., Halaas J.L., Friedman J.M. and Charron M.J. (1997) Acute stimulation of glucose metabolism in mice by leptin treatment. Nature 389, 374–377 10.1038/38717 9311777

[125] da Silva A.A., Hall J.E., Moak S.P., Browning J., Houghton H.J., Micheloni G.C. et al. (2017) Role of autonomic nervous system in chronic CNS-mediated antidiabetic action of leptin. Am. J. Physiol. Endocrinol. Metab. 312, E420–E428 10.1152/ajpendo.00301.2016 27923809 PMC5451526

[126] Perry R.J., Zhang X.M., Zhang D., Kumashiro N., Camporez J.P., Cline G.W. et al. (2014) Leptin reverses diabetes by suppression of the hypothalamic–pituitary–adrenal axis. Nat. Med. 20, 759–763 10.1038/nm.3579 24929951 PMC4344321

[127] Perry R.J., Petersen K.F. and Shulman G.I. (2016) Pleotropic effects of leptin to reverse insulin resistance and diabetic ketoacidosis. Diabetologia 59, 933–937 10.1007/s00125-016-3909-4 26961503 PMC4826798

[128] da Silva A.A., Hall J.E. and do Carmo J.M. (2017) Leptin reverses hyperglycemia and hyperphagia in insulin deficient diabetic rats by pituitary-independent central nervous system actions. PloS ONE 12, e0184805 10.1371/journal.pone.0184805 29190687 PMC5708697

[129] Morton G.J., Meek T.H., Matsen M.E. and Schwartz M.W. (2015) Evidence against hypothalamic-pituitary-adrenal axis suppression in the antidiabetic action of leptin. J. Clin. Invest. 125, 4587–4591 10.1172/JCI82723 26529250 PMC4665796

[130] Morgan D.A., McDaniel L.N., Yin T., Khan M., Jiang J., Acevedo M.R. et al. (2015) Regulation of glucose tolerance and sympathetic activity by MC4R signaling in the lateral hypothalamus. Diabetes 64, 1976–1987 10.2337/db14-1257 25605803 PMC4439564

[131] Obici S., Feng Z., Tan J., Liu L., Karkanias G. and Rossetti L. (2001) Central melanocortin receptors regulate insulin action. J. Clin. Invest. 108, 1079–1085 10.1172/JCI200112954 11581309 PMC200952

[132] Sadagurski M., Leshan R.L., Patterson C., Rozzo A., Kuznetsova A., Skorupski J. et al. (2012) IRS2 signaling in LepR-b neurons suppresses FoxO1 to control energy balance independently of leptin action. Cell. Metab. 15, 703–712 10.1016/j.cmet.2012.04.011 22560222 PMC3361909

[133] Dubinion J.H., do Carmo J.M., Adi A., Hamza S., da Silva A.A. and Hall J.E. (2013) Role of proopiomelanocortin neuron Stat3 in regulating arterial pressure and mediating the chronic effects of leptin. Hypertension 61, 1066–1074 10.1161/HYPERTENSIONAHA.111.00020 23529161 PMC3678380

[134] da Silva A.A., Freeman J.N., Hall J.E. and do Carmo J.M. (2018) Control of appetite, blood glucose, and blood pressure during melanocortin-4 receptor activation in normoglycemic and diabetic NPY-deficient mice. Am. J. Physiol. Regul. Integr. Comp. Physiol. 314, R533–R539 10.1152/ajpregu.00293.2017 29351428 PMC5966815

[135] Xu J., Bartolome C.L., Low C.S., Yi X., Chien C.H., Wang P. et al. (2018) Genetic identification of leptin neural circuits in energy and glucose homeostases. Nature 556, 505–509 10.1038/s41586-018-0049-7 29670283 PMC5920723

[136] Goncalves G.H., Li W., Garcia A.V., Figueiredo M.S. and Bjorbaek C. (2014) Hypothalamic agouti-related peptide neurons and the central melanocortin system are crucial mediators of leptin’s antidiabetic actions. Cell. Rep. 7, 1093–1103 10.1016/j.celrep.2014.04.010 24813890 PMC4369586

[137] da Silva A.A., Hall J.E., Dai X., Wang Z., Salgado M.C. and do Carmo J.M. (2021) Chronic antidiabetic actions of leptin: evidence from parabiosis studies for a CNS-Derived circulating antidiabetic factor. Diabetes 70, 2264–2274 10.2337/db21-0126 34344788 PMC8576509

[138] Mirzadeh Z., Morton G.J., Hirsch I.B. and Schwartz M.W. (2025) An unexpected role for the brain in the pathogenesis of diabetic ketoacidosis. J. Clin. Invest. 135, e196357 10.1172/JCI196357 40759579 PMC12321401

[139] do Carmo J.M., Hall J.E. and da Silva A.A. (2008) Chronic central leptin infusion restores cardiac sympathetic-vagal balance and baroreflex sensitivity in diabetic rats. Am. J. Physiol. Heart Circ. Physiol. 295, H1974–H1981 10.1152/ajpheart.00265.2008 18790839 PMC2614566

[140] Yang R. and Barouch L.A. (2007) Leptin signaling and obesity: cardiovascular consequences. Circ. Res. 101, 545–559 10.1161/CIRCRESAHA.107.156596 17872473

[141] Koh K.K., Park S.M. and Quon M.J. (2008) Leptin and cardiovascular disease: response to therapeutic interventions. Circulation 117, 3238–3249 10.1161/CIRCULATIONAHA.107.741645 18574061 PMC2746068

[142] Packer M. (2026) Do obesity and visceral adiposity promote heart failure with reduced ejection fraction? Eur. Heart J. 47, 12–21 10.1093/eurheartj/ehaf645 40891153 PMC12765561

[143] Theodorakis N. and Nikolaou M. (2025) Leptin and heart failure: the chicken or the egg? Heart Fail. Rev. 30, 749–757 10.1007/s10741-025-10501-6 40090991

[144] Korczynska J., Czumaj A., Chmielewski M., Swierczynski J. and Sledzinski T. (2021) The causes and potential injurious effects of elevated serum leptin levels in chronic kidney disease patients. Int. J. Mol. Sci. 22, 4685 10.3390/ijms22094685 33925217 PMC8125133

[145] Sloan C., Tuinei J., Nemetz K., Frandsen J., Soto J., Wride N. et al. (2011) Central leptin signaling is required to normalize myocardial fatty acid oxidation rates in caloric-restricted ob/ob mice. Diabetes 60, 1424–1434 10.2337/db10-1106 21441440 PMC3292315

[146] Barouch L.A., Berkowitz D.E., Harrison R.W., O’Donnell C.P. and Hare J.M. (2003) Disruption of leptin signaling contributes to cardiac hypertrophy independently of body weight in mice. Circulation 108, 754–759 10.1161/01.CIR.0000083716.82622.FD 12885755

[147] Hall M.E., Harmancey R. and Stec D.E. (2015) Lean heart: role of leptin in cardiac hypertrophy and metabolism. World J. Cardiol. 7, 511–524 10.4330/wjc.v7.i9.511 26413228 PMC4577678

[148] Hall M.E., Smith G., Hall J.E. and Stec D.E. (2012) Cardiomyocyte-specific deletion of leptin receptors causes lethal heart failure in Cre-recombinase-mediated cardiotoxicity. Am. J. Physiol. Regul. Integr. Comp. Physiol. 303, 12, R1241–R1250 10.1152/ajpregu.00292.2012 23115124 PMC3532590

[149] Hall M.E., Maready M.W., Hall J.E. and Stec D.E. (2014) Rescue of cardiac leptin receptors in db/db mice prevents myocardial triglyceride accumulation. Am. J. Physiol. Endocrinol. Metab. 307, E316–E325 10.1152/ajpendo.00005.2014 24939734 PMC4121577

[150] Nguyen M.L., Sachdev V., Burklow T.R., Li W., Startzell M., Auh S. et al. (2021) Leptin attenuates cardiac hypertrophy in patients with generalized lipodystrophy. J. Clin. Endocrinol. Metab. 106, e4327–e4339 10.1210/clinem/dgab499 34223895 PMC8530723

[151] Lee H.L., Waldman M.A., Auh S., Balow J.E., Cochran E.K., Gorden P. et al. (2019) Effects of metreleptin on proteinuria in patients with lipodystrophy. J. Clin. Endocrinol. Metab. 104, 4169–4177 10.1210/jc.2019-00200 30990519 PMC6688455

[152] Brown R.J., Valencia A., Startzell M., Cochran E., Walter P.J., Garraffo H.M. et al. (2018) Metreleptin-mediated improvements in insulin sensitivity are independent of food intake in humans with lipodystrophy. J. Clin. Invest. 128, 3504–3516 10.1172/JCI95476 29723161 PMC6063484

[153] Omoto A.C.M., do Carmo J.M., Mouton A.J., Wang Z., Li X., Spitz R. et al. (2024) Targeting the brain leptin–melanocortin pathway to treat heart failure. Curr. Hypertens. Rep. 27, 2 10.1007/s11906-024-01318-z 39612121 PMC11607000

[154] Gava F.N., da Silva A.A., Dai X., Harmancey R., Ashraf S., Omoto A.C.M. et al. (2021) Restoration of cardiac function after myocardial infarction by long-term activation of the CNS leptin–melanocortin system. JACC Basic Transl. Sci. 6, 55–70 10.1016/j.jacbts.2020.11.007 33532666 PMC7838051

[155] Omoto A.C.M., do Carmo J.M., Nelson B., Aitken N., Dai X., Moak S. et al. (2022) Central nervous system actions of leptin improve cardiac function after ischemia–reperfusion: roles of sympathetic innervation and sex differences. J. Am. Heart Assoc. 11, e027081 10.1161/JAHA.122.027081 36300667 PMC9673649

[156] Lopaschuk G.D. (2021) Targeting the brain to protect the heart. JACC Basic Transl. Sci. 6, 71–73 10.1016/j.jacbts.2020.12.004 33533757 PMC7838095

[157] Omoto A.C.M., Vechetti I., do Carmo J.M., Wang Z., Mouton A.J., Young J.C. et al. (2026) Leptin activates brain–BAT–heart crosstalk to promote cardiac protection. Circ. Res. 138, e326878 10.1161/CIRCRESAHA.125.326878 41636042 PMC12875649

[158] Becher T., Palanisamy S., Kramer D.J., Eljalby M., Marx S.J., Wibmer A.G. et al. (2021) Brown adipose tissue is associated with cardiometabolic health. Nat. Med. 27, 58–65 10.1038/s41591-020-1126-7 33398160 PMC8461455

[159] Starling S. (2021) Human BAT linked with cardiometabolic health. Nat. Rev. Endocrinol. 17, 132 10.1038/s41574-021-00469-2 33462401

[160] do Carmo J.M., Hall J.E., Furukawa L.N.S., Woronik V., Dai X., Ladnier E. et al. (2024) Chronic central nervous system leptin administration attenuates kidney dysfunction and injury in a model of ischemia/reperfusion-induced acute kidney injury. Am. J. Physiol. Renal. Physiol. 327, F957–F966 10.1152/ajprenal.00158.2024 39361725 PMC11687842

[161] Giuliani D., Mioni C., Altavilla D., Leone S., Bazzani C., Minutoli L. et al. (2006) Both early and delayed treatment with melanocortin 4 receptor-stimulating melanocortins produces neuroprotection in cerebral ischemia. Endocrinology 147, 1126–1135 10.1210/en.2005-0692 16254026

[162] Silva A.A., Kuo J.J., Tallam L.S., Liu J. and Hall J.E. (2006) Does obesity induce resistance to the long-term cardiovascular and metabolic actions of melanocortin 3/4 receptor activation? Hypertension 47, 259–264 10.1161/01.HYP.0000198458.70351.e0 16380516

[163] Enriori P.J., Evans A.E., Sinnayah P., Jobst E.E., Tonelli-Lemos L., Billes S.K. et al. (2007) Diet-induced obesity causes severe but reversible leptin resistance in arcuate melanocortin neurons. Cell. Metab. 5, 181–194 10.1016/j.cmet.2007.02.004 17339026

[164] Kuhnen P., Krude H. and Biebermann H. (2019) Melanocortin-4 receptor signalling: importance for weight regulation and obesity treatment. Trends Mol. Med. 25, 136–148 10.1016/j.molmed.2018.12.002 30642682

